# Astragaloside IV attenuates high-glucose-Induced peritoneal fibrosis via modulation of the ENKUR/PI3K/Akt signalling pathway

**DOI:** 10.1371/journal.pone.0348762

**Published:** 2026-05-08

**Authors:** Tianxin Jiang, Lijie Zhang, Jiahan Liu, Xinxin Xu, Yuanyuan Shi, Zhanzheng Zhao, Jing Xiao

**Affiliations:** Department of Nephrology, The First Affiliated Hospital of Zhengzhou University, Zhengzhou, China; Guangdong Nephrotic Drug Engineering Technology Research Center, Institute of Consun Co. for Chinese Medicine in Kidney Diseases, CHINA

## Abstract

This study aimed to explore the mechanisms by which Astragaloside IV (AS-IV), a major bioactive component of *Astragalus membranaceus*, mitigates high-glucose-induced peritoneal fibrosis (PF) in peritoneal dialysis (PD). Using both in vivo (uremic rat model) and in vitro (human peritoneal mesothelial cells) approaches, we observed that AS-IV treatment was associated with a significant attenuation of PF. This effect was mediated through the inhibition of epithelial-mesenchymal transition (EMT) and fibrosis. In vivo, AS-IV reduced extracellular matrix deposition and collagen accumulation, downregulated EMT and fibrosis markers (α-SMA, collagen IV), and restored E-cadherin levels. Notably, these changes correlated with the downregulation of ENKUR, pPI3K, and pAkt. The in vitro results corroborated these findings, showing that AS-IV suppressed EMT without cytotoxic effects. Our data indicate that AS-IV may exert antifibrotic effects via modulation of the ENKUR/PI3K/Akt signaling pathway, suggesting a potential target for the prevention of PF.

## Introduction

Chronic kidney disease (CKD) represents constitutes a growing global health burden crisis, affecting over 10% of the global population and contributing significantly to mortality [1]. Peritoneal dialysis (PD), which utilizes the peritoneum as a semi-permeable membrane，is a vital renal replacement therapy for end-stage CKD patients [2]. However, prolonged exposure to bioincompatible, hyperosmolar glucose-containing dialysates, compounded by factors such as peritoneal hypoxia, uremic toxins, and chronic inflammation, triggers the development of peritoneal fibrosis (PF) – a severe complication that compromises dialysis efficacy and threatens patient survival [3]. Currently, effective clinical interventions for PF are limited.

Pathologically, PF is characterized by angiogenesis, inflammatory infiltration, and the loss of mesothelial cells via epithelial-to-mesenchymal transition (EMT) [3–6]. During EMT, peritoneal mesothelial cells lose epithelial markers (e.g., E-cadherin) and acquire mesenchymal markers (e.g., α-SMA), transforming into fibroblast-like cells that overproduce extracellular matrix, directly driving fibrosis. Numerous canonical signaling pathways, including TGF-β1/Smad, Wnt/β-catenin, and ERK/NF-κB, have been implicated in the progression of EMT. Among these, the PI3K/Akt pathway serves as a critical signaling node in this fibrotic process [7].

ENKUR, an adapter protein located on chromosome 10, plays a pivotal role in Ca² ⁺ -dependent signaling by interacting with ion channels [8]. Structurally, ENKUR contains three functional domains: a C-terminal channel interaction motif, an IQ domain associated with Ca^2+^ sensor, and a proline-rich N-terminus capable of binding the p85 regulatory subunit of PI3K [8]. While ENKUR has been linked to EMT in certain cancers [9–12], its role in PF remains unclear. Preliminary data from our group indicated that ENKUR might play a critical role in the progression of PF.

Astragaloside IV (AS-IV), the primary bioactive component of *Astragalus membranaceus*, exhibits pleiotropic biological activities including anti-inflammatory, antioxidant, and antitumor properties [13]. Accumulating evidence highlights the therapeutic potential of AS-IV in mitigating organ fibrosis, particularly in pulmonary and renal contexts [14,15]. While preclinical studies suggest efficacy in PD models [16–18], the precise molecular mechanisms governing its action remain largely elusive. Notably, to date, no research has explored the potential interaction between AS-IV and ENKUR in the context of PF.

Therefore, this study aims to elucidate whether AS-IV mitigates high-glucose-induced PF through the regulation of the ENKUR/PI3K/Akt pathway.

## Materials and methods

### Materials and reagents

1. **Animals**: Six-week-old male Sprague-Dawley (SD) rats (body weight: 180–200 g) were obtained from the Laboratory Animal Center of Zhengzhou University. Animals were maintained under specific pathogen-free (SPF) conditions in a controlled environment (temperature and humidity) with a 12-h light/dark cycle. Standard rodent chow and water were provided ad libitum.
2. **Cells**: The human peritoneal mesothelial cell line HMrSV5 was acquired from the American Type Culture Collection (ATCC). Cells between passages 6 and 10 (P6–P10) were cultured and employed in this study.
3. **Reagents**: AS-IV was purchased from Nanjing Jingzhu Biotechnology (Catalog No. Z100023). 0.9% physiological saline, dimethyl sulfoxide (DMSO) from Beijing Solarbio (Catalog No. D8371), high-efficiency RIPA lysis buffer for tissues/cells from Beijing Solarbio (Catalog No. R0010), PMSF (100 mM) from Beijing Solarbio (Catalog No. P0100), all-in-one protein phosphatase inhibitor cocktail from Beijing Solarbio (Catalog No. P1260), BCA Protein Assay Kit from Beijing Solarbio (Catalog No. PC0020), PAGE Gel Rapid Preparation Kit from Shanghai Yamei Biotech (Catalog No. PG112), 180 kDa Prestained Protein Marker from Nanjing Vazyme (Catalog No. MP102), 100x 10 × SDS-PAGE Running Buffer from Beijing Bonmaker (Catalog No. PA118–01), ice-free rapid transfer buffer from Wuhan Servicebio (Catalog No. G2154-1L), protein-free rapid blocking solution from Shanghai Yamei Biotech (Catalog No. PS108P), hematoxylin-eosin (H&E) staining kit from Wuhan Servicebio (Catalog No. G1076-500ML), Masson’s trichrome staining kit from Wuhan Servicebio (Catalog No. G1006), S-vision IHC kit from Wuhan Servicebio (Catalog No. G1313-100T), ENKUR antibody (Enkurin Polyclonal antibody) from Wuhan Proteintech (Catalog No. 26440–1-AP), α-SMA antibody (Smooth muscle actin Polyclonal antibody) from Wuhan Proteintech (Catalog No. 14395–1-AP), E-cadherin antibody (E-cadherin Polyclonal antibody) from Wuhan Proteintech (Catalog No. 20874–1-AP), Collagen IV antibody (Collagen Type IV Polyclonal antibody) from Wuhan Proteintech (Catalog No. 19674–1-AP), Phospho-PI3 Kinase p85 (Tyr458)/p55 (Tyr199) Rabbit mAb from Cell Signaling Technology (Catalog No. 17366T), PI3K antibody (PI3 Kinase p85 Alpha Monoclonal antibody) from Wuhan Proteintech (Catalog No. 60225–1-Ig), Phospho-AKT (Ser473) antibody from Wuhan Proteintech (Catalog No. 66444–1-Ig), AKT antibody from Wuhan Proteintech (Catalog No. 60203–2-Ig), GAPDH antibody from Wuhan Proteintech (Catalog No. 60004–1-Ig), β-Actin antibody from Wuhan Proteintech (Catalog No. 20536–1-AP), HRP-conjugated Goat Anti-Rabbit IgG(H + L) secondary antibody from Wuhan Proteintech (Catalog No. SA00001–2), HRP-conjugated Goat Anti-Mouse IgG(H + L) secondary antibody from Wuhan Proteintech (Catalog No. SA00001–1), Western primary antibody dilution buffer from Shanghai Beyotime (Catalog No. P0023A), SuperKine™ Ultra-sensitive ECL substrate from Wuhan AcoBiotech (Catalog No. BMU102), Dulbecco’s Modified Eagle Medium (DMEM) from Gibco (Catalog No. 11885084), fetal bovine serum from Uruguay GoldORG (Catalog No. E600051-0500), Trypsin-EDTA without phenol red from Beijing Solarbio (Catalog No. T1300), CCK-8 Cell Proliferation and Cytotoxicity Assay Kit from Wuhan AcoBiotech (Catalog No. KTA1020), SweScript All-in-One RT SuperMix for qPCR (One-Step gDNA Remover) kit from Wuhan Servicebio (Catalog No. G3337-50), and Taq Pro Universal SYBR qPCR Master Mix from Nanjing Vazyme (Catalog No. Q712).

### Experimental methods

1. **Construction of a 5/6 Nephrectomy Uremic PD Rat Model**: (1) Animal Grouping: Male SD rats were randomly assigned to four groups (n = 6/group) using a random number table: a. Sham: Bilateral renal capsule exposure only. b. Uremia (5/6 Nx): Underwent 5/6 nephrectomy (5/6 Nx). c. PD: 5/6 Nx + PD. d. AS-IV: 5/6 Nx + PD + AS-IV. (2) Model Construction: a. Sham: Rats underwent bilateral flank incisions with renal capsule exposure only. b. Uremia: Rats underwent subtotal (5/6) nephrectomy. c. PD: Six weeks post-5/6 Nx, rats underwent PD catheter implantation. The rats received 4.25% glucose-based PD solution (3 mL/100g body weight/day) supplemented with DMSO solvent (40 mg/kg/day; 0.1% v/v) for 4 weeks. d. AS-IV: In addition to PD treatment (as above), rats received daily intraperitoneal injections of AS-IV (40 mg/kg/day [17,19] dissolved in DMSO, 0.1% v/v) for 4 weeks. At the experimental endpoint, all rats underwent a standardized 4-hour peritoneal equilibration test (PET): each rat underwent intraperitoneal infusion of 20 mL of 4.25% glucose-based PD solution. After a standardized 4-hour dwell period, the rats were euthanized, blood samples were collected, and the dialysate was quantitatively collected to determine the ultrafiltration volume. Both the baseline (pre-infusion) PD solution and the 4-hour effluent samples were aliquoted and preserved for subsequent biochemical analyses. Peritoneal tissue was harvested; portions were fixed in 4% paraformaldehyde for paraffin embedding, and the remainder was snap-frozen in liquid nitrogen for subsequent analysis. This study was carried out in strict accordance with the recommendations in the Guide for the Care and Use of Laboratory Animals of the National Institutes of Health (2023-KY-1070–002). The protocol was approved by the Ethics Committee of the First Affiliated Hospital of Zhengzhou University, Zhengzhou University, China (2023-KY-1344). All surgery was performed under sodium pentobarbital anesthesia, and all efforts were made to minimize suffering.
2. **Biochemical Analysis of Rat Samples**: Peritoneal effluent and blood samples collected from all experimental groups were immediately transferred to 2 mL centrifuge tubes and held at ambient temperature. Following centrifugation at 3,000 × g for 15 min, supernatants were aspirated. Aliquots were subsequently transferred to the Nephrology Laboratory, First Affiliated Hospital of Zhengzhou University, for biochemical analysis. Key analytes included serum creatinine and glucose concentrations in peritoneal effluent at 0 h (initial dwell) and 4 h (post-equilibration test).
3. **Hematoxylin and Eosin (HE) and Masson’s Trichrome Staining**: Following euthanasia, peritoneal tissue was harvested from all rats. Full-thickness sections (~1 cm²) were immediately immersed in 4% paraformaldehyde and fixed at 4°C overnight. Tissues underwent sequential dehydration, clearing, paraffin embedding, and sectioning. HE and Masson’s trichrome staining were performed on 4-μm sections to assess pathological alterations in peritoneal morphology, including collagen deposition and submesothelial thickening.
4. **Immunohistochemistry**: Deparaffinized and rehydrated sections underwent heat-mediated antigen retrieval. Endogenous peroxidase activity was quenched with 3% H₂O₂ (15 min), followed by blocking with 10% normal goat serum (30 min). Sections were incubated overnight at 4°C with primary antibodies: α-SMA (dilution 1:1500) and ENKUR (dilution 1:150). After PBS washes, sections were treated with HRP-conjugated secondary antibody (ABC Kit, Vector Laboratories) for 1 h at RT. Chromogenic detection used DAB substrate (5 min), with hematoxylin counterstaining. Sections were dehydrated, cleared in xylene, and mounted for brightfield microscopy.
5. **Cell Culture**: Cryopreserved HMrSV5 cells were rapidly thawed in a 37°C water bath, resuspended in 3 mL complete growth medium (DMEM + 10% FBS), and centrifuged (300 × *g*, 5 min, 4°C). After supernatant removal, the pellet was reconstituted in fresh growth medium. Cells were maintained at 37°C/5% CO₂ with medium replacement every 48–72 h. At 80–90% confluence, subculture was performed using 0.25% trypsin-EDTA (2–3 min incubation). Cells in logarithmic growth phase (24–48 h post-seeding) were utilized for experiments.
6. **Cell Viability Assay**: At the logarithmic growth phase, HMrSV5 cells were seeded in 96-well plates (3.5 × 10⁴ cells/well) and incubated for 24 h. Experimental groups received AS-IV (10−100 μg/mL; 10,20,30,50,80,100 μg/mL, respectively) for 24 h, while controls received vehicle (0.1% DMSO v/v). Cell viability was quantified using the CCK-8 assay per the manufacturer’s protocol.
7. **Cell Models**: (1) High Glucose Stimulation Model: Grouping: a. NG: Normal glucose (5.56 mM). b. NG + M: Normal glucose + 25 mM mannitol (osmotic control). c. 1.5%: 1.5% glucose (w/v, equivalent to 83.3 mM). d. 2.5%: 2.5% glucose (w/v, equivalent to 138.9 mM). HMrSV5 cells underwent 48 h exposure under respective conditions. (2) AS-IV Intervention Model: Cells pretreated with 2.5% glucose + 0.1% DMSO (24 h) were grouped as: a. HG: Vehicle control (continued DMSO). b. 10: 10 μg/mL AS-IV. c. 30: 30 μg/mL AS-IV. d. 50: 50 μg/mL AS-IV. Interventions lasted 24 h.
8. **Western Blot**: Total protein was extracted from tissues and cells using ice-cold RIPA lysis buffer. Lysates were centrifuged (12,000 × *g*, 15 min, 4°C), and supernatants collected for quantification via BCA assay. Proteins (15 μg/lane) were resolved by SDS-PAGE and transferred to PVDF membranes. After blocking with protein-free buffer (30 min, RT), membranes were incubated overnight at 4°C with primary antibodies: ENKUR (1:3000), α-SMA (1:3000), E-cadherin (1:10000), Collagen IV (1:3000), P-PI3K (1:1000), PI3K (1:5000), P-AKT (1:5000), AKT (1:5000), GAPDH (1:50000), and β-Actin (1:5000). Following TBST washes (3 × 10 min), membranes were probed with HRP-conjugated secondary antibodies (1:5,000, 1 h, RT). After additional TBST washes, proteins were detected using enhanced chemiluminescence. β-Actin and GAPDH served as loading controls. Band intensities were quantified with ImageJ.
9. **Quantitative Real-Time PCR (qRT-PCR)**: Total RNA was isolated using TRIzol reagent, with concentration and purity assessed spectrophotometrically (A260/A280). cDNA was synthesized from 900 ng RNA using a reverse transcription kit (PrimeScript RT, Takara). qPCR reactions contained 2 μL cDNA template and were performed in triplicate using SYBR Green Master Mix (Roche) under cycling conditions: 95°C/30 sec initial denaturation; 40 cycles of 95°C/10 sec, 60°C/30 sec; fluorescence acquisition during annealing. GAPDH served as an endogenous control. Relative mRNA expression was calculated by the 2 − ΔΔCT method, where ΔΔCT = [CT(target gene in experimental group) – CT(reference gene in experimental group)] – [CT(target gene in control group) – CT(reference gene in control group)]. The CT value is the amplification cycle number determined from the amplification curve. Gene-specific primers (Shangya Biotechnology, China) are listed in Table 1.

**Table 1:** Primer sequences.

Genes	Forward primer	Reverse primer
GAPDH-Homo	GGCATCCTGGGCTACACT	CCACCACCCTGTTGCTGTA
ENKUR-Homo	CCAGTTCAACCTCCCCCAAT	GGGGCCAGACCACATCAAAT
α-SMA-Homo	CCTGACTGAGCGTGGCTATT	GCCCATCAGGCAACTCGTAA
E-cadherin-Homo	ACCATTAACAGGAACACAGG	CAGTCACTTTCAGTGTGGTG
Collagen IV-Homo	CACGGACAAGACCTTGGAACTC	GGTGTTGACAGCCAGTATGAATAATC
GAPDH-Rat	AAGTTCAACGGCACAGTCAAGG	GACATACTCAGCACCAGCATCAC
ENKUR-Rat	GTTCCAGTCCCTCTCGGTCTTC	TTGTGCTTCTCGATGATGCTGATG
α-SMA-Rat	AGGGAGTGATGGTTGGAATGGG	GGTGATGATGCCGTGTTCTATCG
E-cadherin-Rat	AGATCAGGACCAGGACTACGATTATC	CTTCGCCGCCACCATACATATC
Collagen IV-Rat	CCGAGCCAGTCCGTTTATAGAATG	GGATTTAGTGAAGCCAGCCAGAAG

### Statistical analysis

Statistical analyses and graph generation were performed using GraphPad Prism 8 software.

All experiments were performed with a minimum of three independent replicates. Data are presented as mean ± standard error of the mean(mean ± SD). Prior to parametric analyses, the normality of distribution was assessed using the Shapiro-Wilk test, and the homogeneity of variances was evaluated with Levene’s test. Statistical comparisons between groups were conducted by one-way analysis of variance (ANOVA) followed by the Least Significant Difference (LSD) post hoc test. A p-value of less than 0.05 was considered statistically significant.

## Results

### AS-IV ameliorates PF and improves peritoneal ultrafiltration function in PD rats

**Fig 1:**
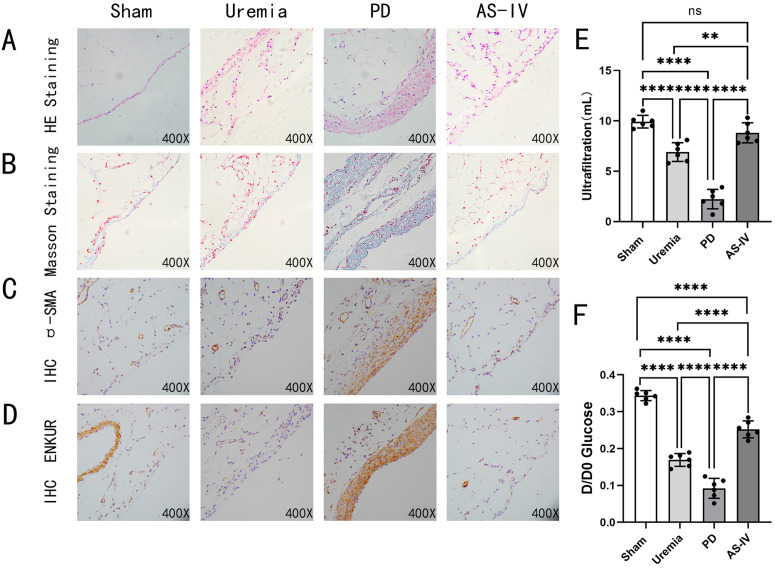
AS-IV ameliorates PF and improves peritoneal ultrafiltration function in PD rats. Peritoneal pathological changes and transport function in vivo. **A** HE staining for general tissue morphology. **B** Masson staining for collagen (blue). **C-D** Immunohistochemical analysis of tissue sections, **C** α-SMA expression, **D** ENKUR expression. **E** The volume of peritoneal dialysate obtained from different groups of rats after a 4 hours of PET. **F** Glucose transport status was assessed using PET at 0 and 4 hours, with D and D0 representing the glucose concentrations in the peritoneal effluent at 0 and 4 hours, respectively. *p < 0.05, **p < 0.01, ***p < 0.001, ****p < 0.0001 for ANOVA, ns, not significant.

To investigate the effects of AS-IV on PF and peritoneal ultrafiltration function in a rat model of PD, male SD rats underwent uremia induction surgery followed by PD to establish the PD rat model. Body weight in the AS-IV group was not significantly affected by intraperitoneal injection of AS-IV compared to the other groups (Supporting Information S8). After 4 weeks of PD, histological analyses were performed to assess peritoneal changes. H&E staining results revealed that the peritoneum in the Sham group was smooth and intact, with a thin submesothelial matrix and a complete layer of mesothelial cells. In contrast, the Uremia group exhibited a slight thickening of the peritoneum, although this difference was not statistically significant. The PD group exhibited significant peritoneal alterations, including mesothelial cell exfoliation, angiogenesis, and marked peritoneal thickening. Notably, AS-IV treatment significantly reduced these structural abnormalities comparing to the PD group (Fig 1A). Masson’s trichrome staining indicated that, compared to the Sham group, there was a slight deposition of collagen fibers in the peritoneum of the Uremia group, although this difference did not reach statistical significance. In contrast, it confirmed a significant increase in collagen fiber deposition in the PD group, which was attenuated by AS-IV(Fig 1B). Immunohistochemical analysis revealed that, compared to the Sham group, there was no significant difference in the expression of ENKUR and the fibroblast marker α-SMA in the peritoneum of the Uremia group. However, it showed elevated expression of the fibroblast marker α-SMA and ENKUR in the PD group compared to the Sham group. AS-IV administration significantly reduced the expression of both proteins (Fig 1C, 1D).

To evaluate peritoneal transport function, a PET was performed. The results showed that, compared to the Sham group, both the Uremia group and the PD group reduced ultrafiltration volume and the D4h glucose/D0h glucose ratio, with the decrease being more significant in the PD group (P < 0.0001). AS-IV treatment partially restored these parameters (P < 0.0001) (Fig 1E, 1F). These findings indicated that AS-IV may ameliorate PF and improve peritoneal ultrafiltration function in rats undergoing PD. The relevant raw data for Fig 1 are available in the Supporting Information (Supporting S4 Fig 1 dataset in S4 File).

### AS-IV alleviates PF in PD rats via the ENKUR/PI3K/Akt pathway

**Fig 2:**
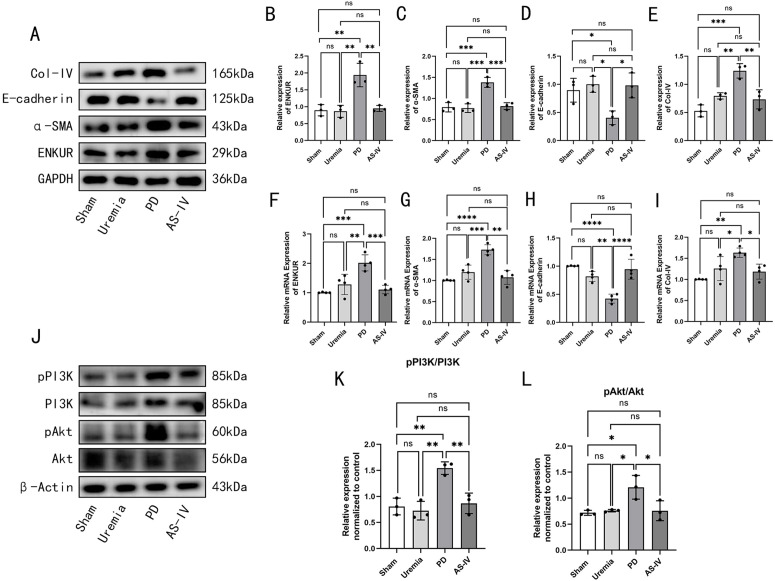
AS-IV alleviates PF in PD rats via the ENKUR/PI3K/Akt pathway. The expression of EMT and fibrosis markers as well as ENKUR/PI3K/Akt in vivo. **A** Western blot images for ENKUR, α-SMA, E-cadherin and Col-IV expression with different treatments. **B–E** Quantitative analysis of the relative expression levels of proteins: **B** ENKUR, **C** α-SMA, **D** E-cadherin, **E** Col-IV. **F-I** Quantitative analysis of the relative expression levels of mRNAs: **F** ENKUR, **G** α-SMA, **H** E-cadherin, **I** Col-IV. **J** Western blot images for PI3K/Akt pathway expression with different treatments. **K–L** Quantitative analysis of the relative expression levels of proteins: **K** pPI3K/PI3K, **L** pAkt/Akt. *p < 0.05, **p < 0.01, ***p < 0.001, ****p < 0.0001 for ANOVA, ns, not significant.

Western blot and qRT-PCR analyses were employed to assess the expression levels of fibrosis markers, the PI3K/Akt pathway activation, and ENKUR in each group of rats. The results indicated that the PD group exhibited significantly upregulated mRNA and protein levels of ENKUR, α-SMA, and Collagen IV, alongside a downregulation of E-cadherin. Furthermore, the phosphorylation levels of PI3K and Akt were markedly increased. AS-IV treatment reversed these trends, significantly downregulating ENKUR and the phosphorylation of PI3K/Akt (P < 0.05) (Fig 2). These findings indicated that AS-IV may alleviate PF in PD rats through the modulation of the ENKUR/PI3K/Akt signaling pathway. The relevant raw data for Fig 2 are available in the Supporting Information (Supporting S5 Fig 2 dataset in S5 File).

### High glucose induces ENKUR expression and EMT in HMrSV5 cells

**Fig 3:**
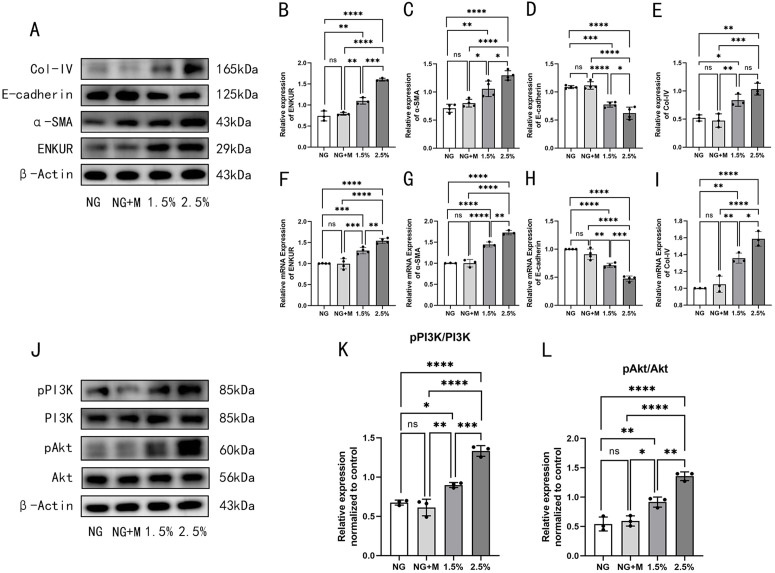
High glucose induces ENKUR expression and EMT in HMrSV5 cells. The expression of EMT and fibrosis markers as well as ENKUR/PI3K/Akt in vitro. **A** Western blot images for ENKUR, α-SMA, E-cadherin and Col-IV expression under high glucose stimulation. **B–E** Quantitative analysis of the relative expression levels of proteins: **B** ENKUR, **C** α-SMA, **D** E-cadherin, **E** Col-IV. **F-I** Quantitative analysis of the relative expression levels of mRNAs: **F** ENKUR, **G** α-SMA, **H** E-cadherin, **I** Col-IV. **J** Western blot images for PI3K/Akt pathway expression under high glucose stimulation. **K–L** Quantitative analysis of the relative expression levels of proteins: **K** pAkt/Akt, **L** pPI3K/PI3K. *p < 0.05, **p < 0.01, ***p < 0.001, ****p < 0.0001 for ANOVA, ns, not significant.

Light microscopy was employed to observe morphological changes in HMrSV5 cells under varying glucose concentrations. Cells in the NG group and the NG + M ones exhibited a characteristic smooth cobblestone morphology with no significant difference. In contrast, cells exposed to 1.5% glucose began to display a spindle-shaped morphology, which became more significant in cells treated with 2.5% glucose. This phenotypic transition was associated with a concentration-dependent upregulation of ENKUR, α-SMA, and Collagen IV, and a downregulation of E-cadherin. Additionally, the phosphorylation of PI3K and Akt was significantly increased (P > 0.05) (Fig 3). The relevant raw data for Fig 3 are available in the Supporting Information (Supporting S6 Fig 3 dataset in S6 File).

### AS-IV alleviates high glucose-induced EMT in HMrSV5 cells via inhibition of the ENKUR/PI3K/Akt pathway

**Fig 4:**
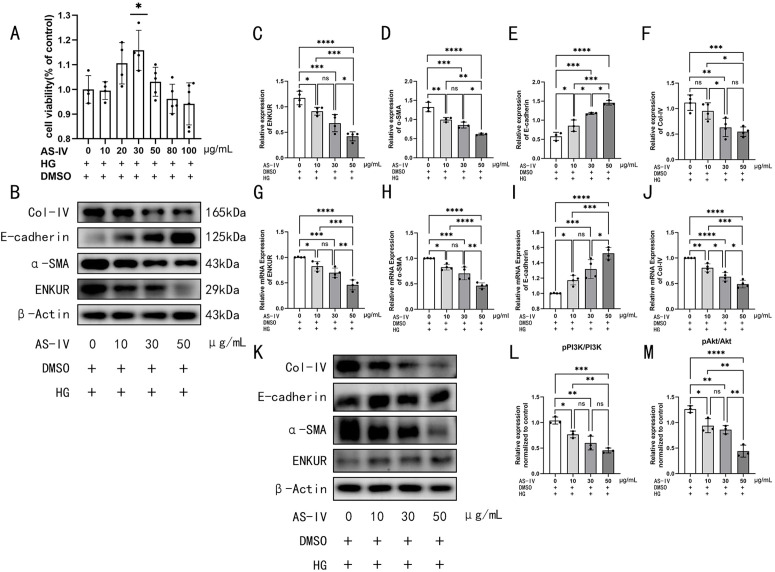
AS-IV alleviates high glucose-induced EMT in HMrSV5 cells via inhibition of the ENKUR/PI3K/Akt pathway. The expression of EMT and fibrosis markers as well as ENKUR/PI3K/Akt in vitro. **A** Cell viability of HMrSV5 treated with different concentrations of AS-IV (0, 10, 20, 30, 50, 80, 100 μg/ml) was assessed in a HG environment containing 2.5% glucose using the CCK8 assay. **B** Western blot images for ENKUR, α-SMA, E-cadherin and Col-IV expression under high glucose stimulation and AS-IV intervention. **C–F** Quantitative analysis of the relative expression levels of proteins: **C** ENKUR, **D** α-SMA, **E** E-cadherin, **F** Col-IV. **G-J** Quantitative analysis of the relative expression levels of mRNAs: **G** ENKUR, **H** α-SMA, **I** E-cadherin, **J** Col-IV. **K** Western blot images for PI3K/Akt pathway expression under high glucose stimulation and AS-IV intervention. **L–M** Quantitative analysis of the relative expression levels of proteins: **L** pPI3K/PI3K, **M** pAkt/Akt. *p < 0.05, **p < 0.01, ***p < 0.001, ****p < 0.0001 for ANOVA, ns, not significant.

The impact of varying AS-IV concentrations on the viability of HMrSV5 cells was evaluated using the CCK-8 assay. The findings indicated that cell viability initially increased and subsequently declined in a concentration-dependent manner as the concentration of AS-IV increased, and AS-IV (30 μg/mL) exhibited optimal cell viability without cytotoxicity (Fig 4A). Treatment with AS-IV (10–50 μg/mL) concentration-dependently suppressed the high glucose-induced upregulation of ENKUR, α-SMA, and Collagen IV, while restoring E-cadherin levels. Concurrently, AS-IV inhibited the phosphorylation of PI3K and Akt in a concentration-dependent way (P < 0.05) (Fig 4). The relevant raw data for Fig 4 are available in the Supporting Information (Supporting S7 Fig 4 dataset in S7 File).

## Discussion

PF, primarily driven by prolonged exposure to high-glucose dialysate, severely compromises peritoneal ultrafiltration capacity and represents a major cause of ultrafiltration failure in PD [3,6,20]. Therefore, identifying effective pharmacological interventions is critical. *Astragalus membranaceus*, a cornerstone of traditional Chinese medicine, and its primary active ingredient, AS-IV, have garnered extensive clinical attention due to their diverse pharmacological properties [21]. Among its constituents, AS-IV [22] has demonstrated therapeutic efficacy in various forms of organ fibrosis [23–26]. Specifically, in the context of PF, Zhang et al. [16] have shown that AS-IV facilitates the upregulation of Smad7 within the TGF-β1/Smad signaling pathway, thereby reversing the EMT of HMrSV5 cells, which underscores its potential therapeutic utility in PF. Moreover, AS-IV has been implicated in ameliorating PF by promoting PGC-1α, which contributes to reduced apoptosis both in vitro and in vivo [17]. Recent investigations have also highlighted its role in attenuating macrophage-derived exosomes-induced fibrosis in PD through the miR–204–5p/Foxc1 pathway [18]. Nonetheless, the precise mechanisms by which AS-IV exerts its effects on PF remain to be fully elucidated.

Our findings indicate that AS-IV effectively attenuates high-glucose-induced PF in a uremic rat model, as evidenced by reduced peritoneal thickening and improved ultrafiltration function. The expression of ENKUR as well as the activation and phosphorylation of the PI3K/Akt pathway were markedly inhibited by AS-IV. In vitro studies revealed the concentration-dependent mitigation of high-glucose-induced EMT in HMrSV5 cells, alongside the inhibition of ENKUR expression and the PI3K/Akt pathway activation. These findings indicate that AS-IV may attenuate EMT and fibrosis in PMCs by suppressing the ENKUR/PI3K/Akt pathway, thereby mitigating high-glucose induced damage to peritoneal structure and function.

Prolonged exposure to biologically incompatible high-glucose PD dialysate can alter the structure and function of the peritoneum [5,27,28]. Prior research has established that high-glucose PD dialysate induces morphological changes in PMCs, transitioning from an epithelial to a fibroblast-like phenotype, accompanied by cell detachment and submesothelial collagen fiber deposition, which are pivotal in PF [5]. Our study observed thickening of the peritoneum, collagen fiber deposition, decreased peritoneal ultrafiltration capacity, and increased expression of fibrosis markers in uremic PD rats. Conversely, the AS-IV group exhibited a marked reduction in peritoneal thickness and improved ultrafiltration function, along with decreased fibrosis. In vitro, high-glucose stimulation prompted PMCs to transition from a cobblestone-like to a spindle-shaped morphology, with downregulation of mesothelial cell markers and upregulation of fibroblast markers. These observations indicate that prolonged exposure to high-glucose PD dialysate can induce EMT in PMCs, leading to the development and progression of PF. Importantly, AS-IV effectively counteracted these pathological changes, mitigating EMT and preserving peritoneal membrane integrity.

Multiple studies have implicated that ENKUR is involved in the EMT process in various cancer cells, where it acts as a tumor suppressor by degrading MYH9 to inhibit EMT signaling and tumor metastasis [9–12]. Our study reveals that high-glucose PD dialysate induces EMT in HPMCs, with elevated ENKUR expression correlating with increased fibrosis markers and functional decline in uremic PD rats. Moreover, our preliminary studies have also shown that silencing ENKUR suppresses activation of the PI3K/Akt pathway, thereby attenuating EMT in PMCs. Notably, AS-IV treatment not only attenuated PF and improved ultrafiltration but also significantly suppressed ENKUR expression. In vitro analyses confirmed that high-glucose-stimulated EMT in PMCs, marked by mesenchymal marker upregulation, was consistently associated with ENKUR elevation. These findings position ENKUR as a potential mediator of peritoneal fibrosis, with its suppression (e.g., by AS-IV) offering a therapeutic avenue for PF intervention.

The PI3K/Akt signaling pathway is a central regulatory pathway for cell growth, metabolism, and survival [29]. It plays a pivotal role in EMT in tumor cells and peritoneal cells. Jia et al. [30] demonstrated that the PI3K/Akt pathway is significantly activated in a high-glucose-induced peritoneal fibrosis rat model, and its inhibition activates autophagy during PD, inhibiting peritoneal EMT and fibrosis. In our study, levels of pPI3K and pAkt we significantly increased in the peritoneal tissue of PD group rats compared to the Sham group. Similarly, the PI3K/Akt pathway was also activated in the high-glucose-treated HPMCs group. Importantly, subsequent experiments in PD rat models and in vitro studies supported that AS-IV may inhibit the activation of the ENKUR/PI3K/Akt pathway, thereby suppressing EMT and fibrosis in PMCs and subsequently mitigating high-glucose -induced damage to peritoneal structure and function.

We then employed databases such as SwissTargetPrediction, STITCH, and STRING to identify potential targets of AS-IV, the PI3K/Akt pathway, and ENKUR. In the protein-protein interaction (PPI) network analysis, we discovered that AS-IV targets three upstream, two core, and two downstream components of the PI3K/Akt pathway. ENKUR interacts with the core component of the PI3K/Akt pathway. Notably, PIK3R1 and Akt1, as upstream and downstream genes within the pathway, have a direct interaction. Gene Ontology (GO) enrichment analysis revealed that the intersection targets of AS-IV and the PI3K/Akt pathway are closely related to cell proliferation and migration, suggesting that AS-IV may improve peritoneal dialysis-related pulmonary fibrosis through these intersecting targets. Building on our previous research indicating that ENKUR participates in the fibrosis and EMT processes induced by high glucose in HMrSV5 cells through its action on the PI3K/Akt pathway, we propose that AS-IV may alleviate high glucose-induced PF by inhibiting the ENKUR/PI3K/Akt signaling pathway.

While our findings suggest a potential association between AS-IV, ENKUR downregulation, and PI3K/Akt inhibition, certain limitations should be acknowledged. The limited number of EMT biomarkers examined in our study may not fully capture the complexity of the EMT process, potentially weakening the robustness of our conclusions. Although we observed a correlation between ENKUR and fibrosis, direct evidence of AS-IV binding to ENKUR requires further validation. Future studies employing molecular docking and direct binding assays (e.g., pull-down) are warranted to determine if ENKUR is a direct molecular target of AS-IV. Besides, comprehensive in vivo dose-response and time-course studies are needed to fully characterize the pharmacokinetic and pharmacodynamic profile of AS-IV in the context of PF. Additionally, recent studies have reported that AS-IV can mitigate doxorubicin-induced cardiomyopathy by inhibiting autophagy [31] and suppress PM2.5-mediated lung injury in mice by regulating ferroptosis signaling via the Nrf2/SLC7A11/GPX4 axis [32]. These programmed cell death mechanisms may also be involved in AS-IV-mediated protection and warrant further investigation.

## Conclusion

In summary, this study provides evidence that AS-IV may attenuate high-glucose-induced EMT and fibrosis, at least in part, by modulating the ENKUR/PI3K/Akt pathway. This study identifies ENKUR as a potential therapeutic target and offers new insights into the molecular mechanisms underlying the therapeutic potential of AS-IV in PF.

## Supporting information

S1 File S1 raw images WB appendix. (DOCX)

S2 File S1 raw images WB. (PDF)

S3 File S2 raw images pathology. (PDF)

S4 File S4 Fig 1 dataset. (XLSX)

S5 File S5 Fig 2 dataset. (XLSX)

S6 File S6 Fig 3 dataset. (XLSX)

S7 File S7 Fig 4 dataset. (XLSX)

S8 File S8 weight of rats. (XLSX)
