# Supplementary material for: Astragaloside IV attenuates high-glucose-Induced peritoneal fibrosis via modulation of the ENKUR/PI3K/Akt signalling pathway
Source: PLoS One. 2026 May 8;21(5):e0348762. doi: 10.1371/journal.pone.0348762 (PMC13155615; doi:10.1371/journal.pone.0348762)
Supplement: S1 File — (DOCX) [file pone.0348762.s001.docx]

The theoretical position of Col-IV is around 165kDa, but the position of COL-IV is always around 130kDa when we actually perform WB. This may be related to electrophoresis conditions and gel concentration. Since it has no excess miscellaneous bands, we temporarily retain the validity of this data.
