# Supplementary material for: Astragaloside IV attenuates high-glucose-Induced peritoneal fibrosis via modulation of the ENKUR/PI3K/Akt signalling pathway
Source: PLoS One. 2026 May 8;21(5):e0348762. doi: 10.1371/journal.pone.0348762 (PMC13155615; doi:10.1371/journal.pone.0348762)
Supplement: S2 File — (PDF) [file pone.0348762.s002.pdf]

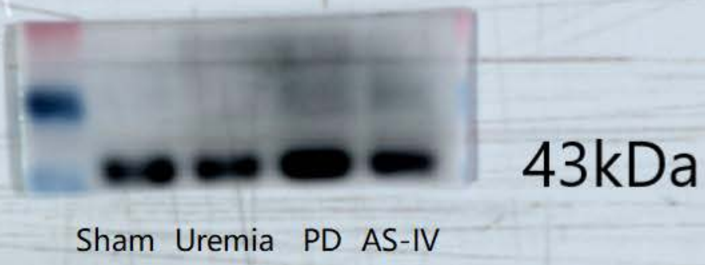

Fig 2A α-SMA

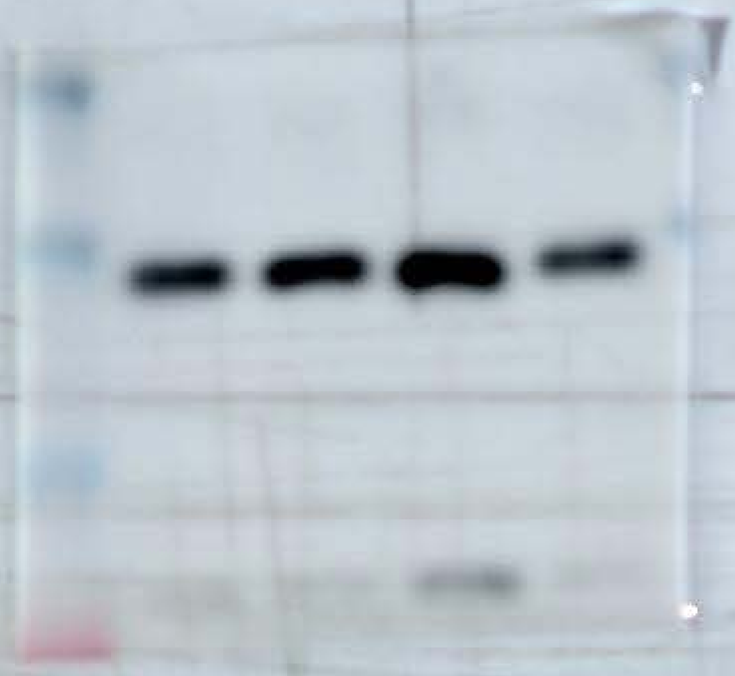

Fig 2A Col-IV

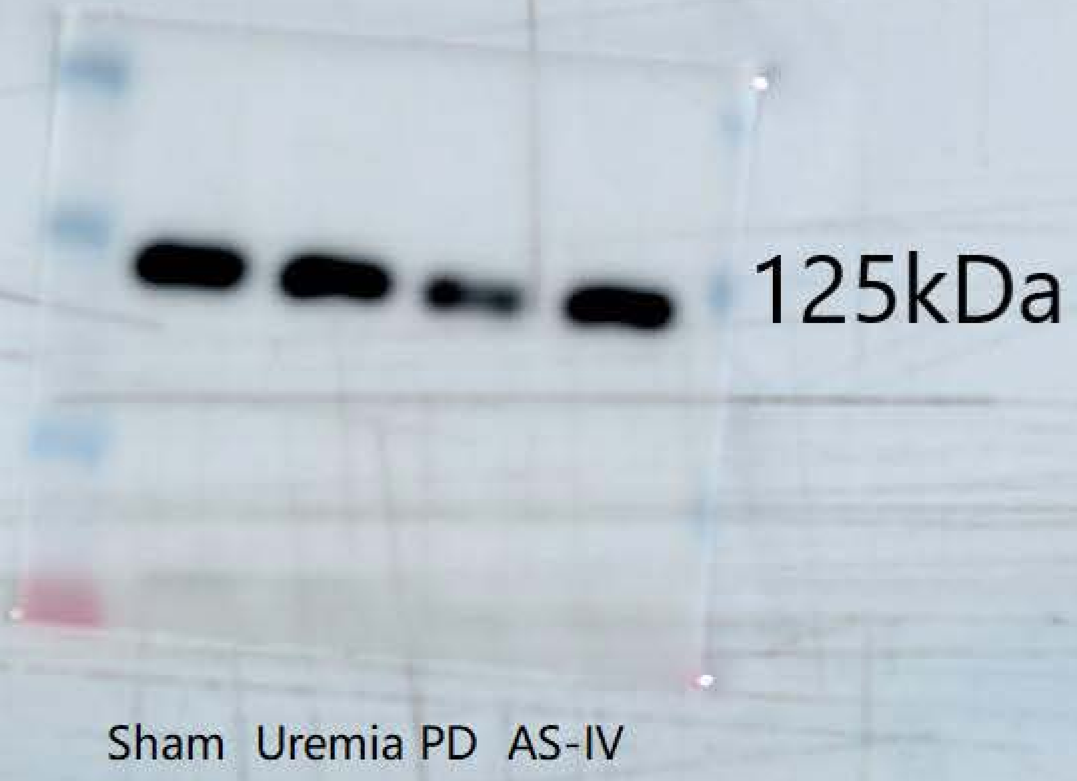

Fig 2A E-cadherin

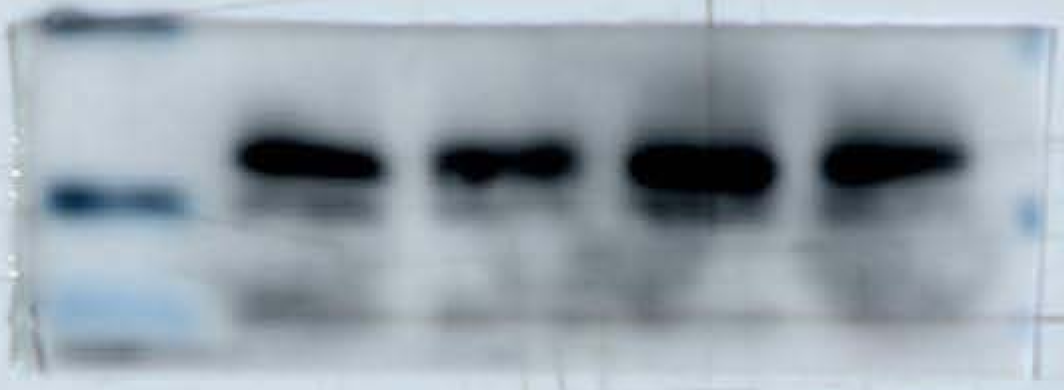

Fig 2A ENKUR

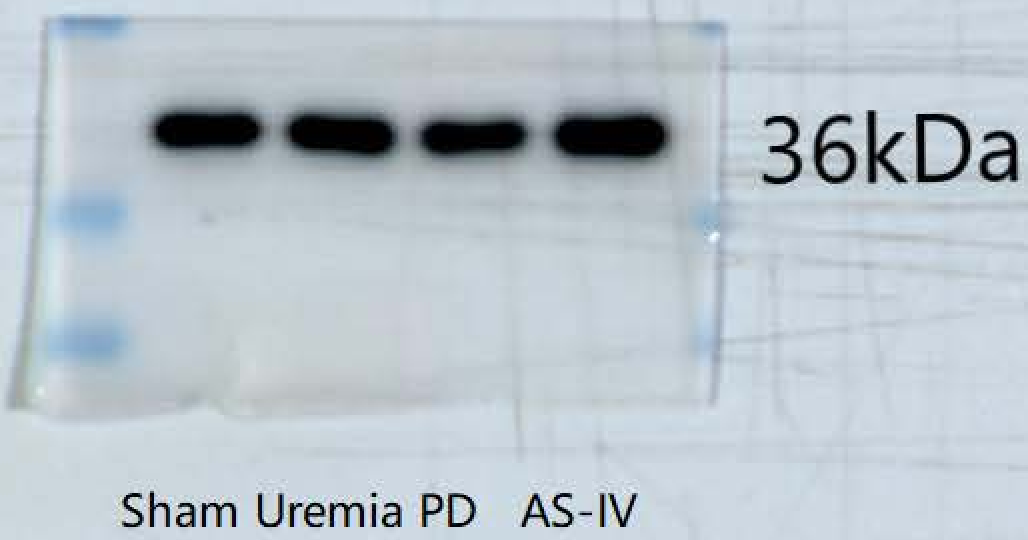

Fig 2A GAPDH

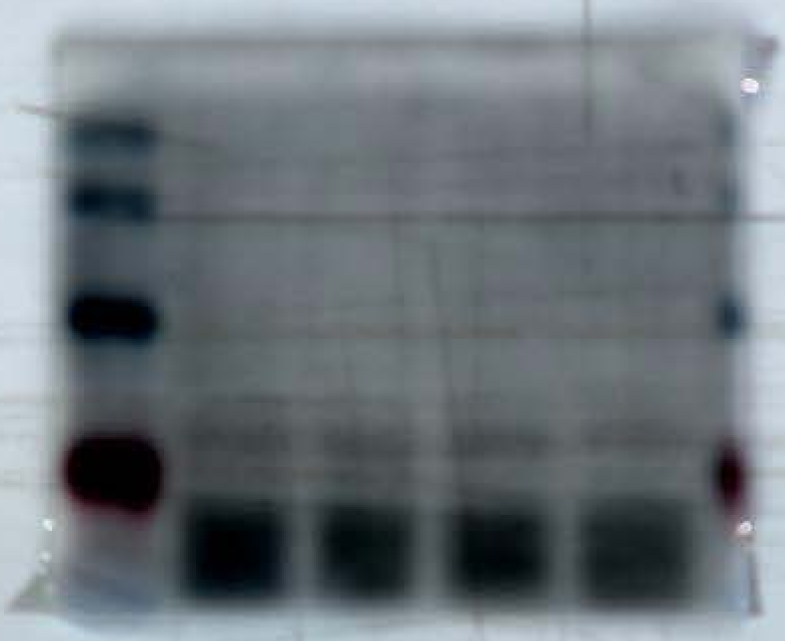

56kDa

Sham Uremia PD AS-IV

Fig 2J AKT

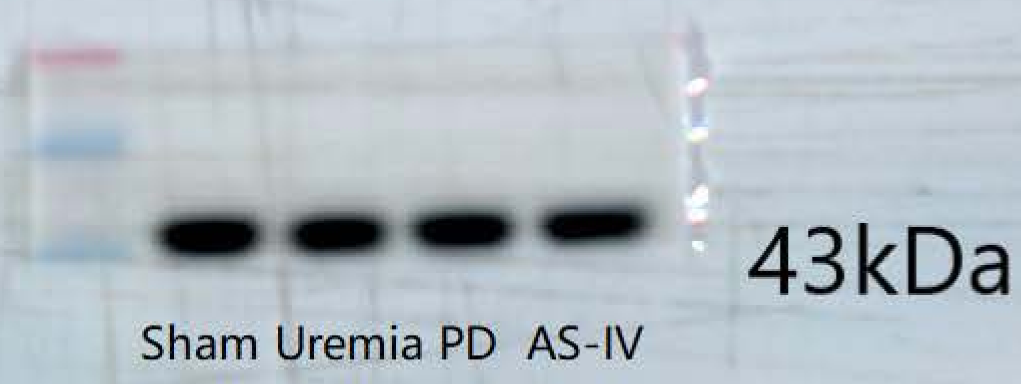

Fig 2J BACTIN

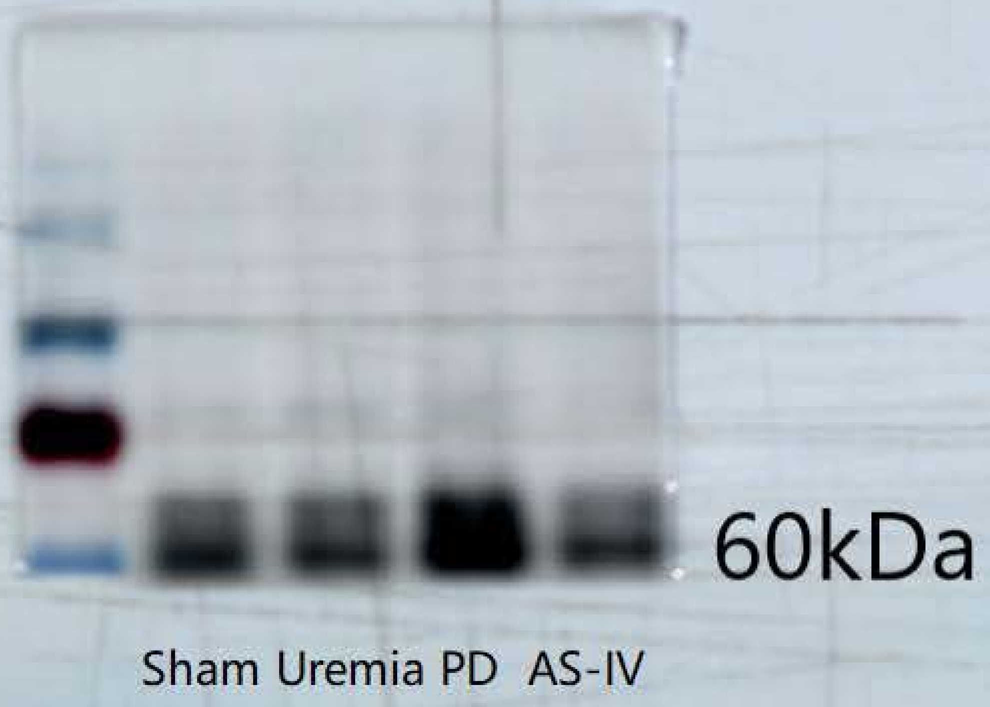

Fig 2J pAKT

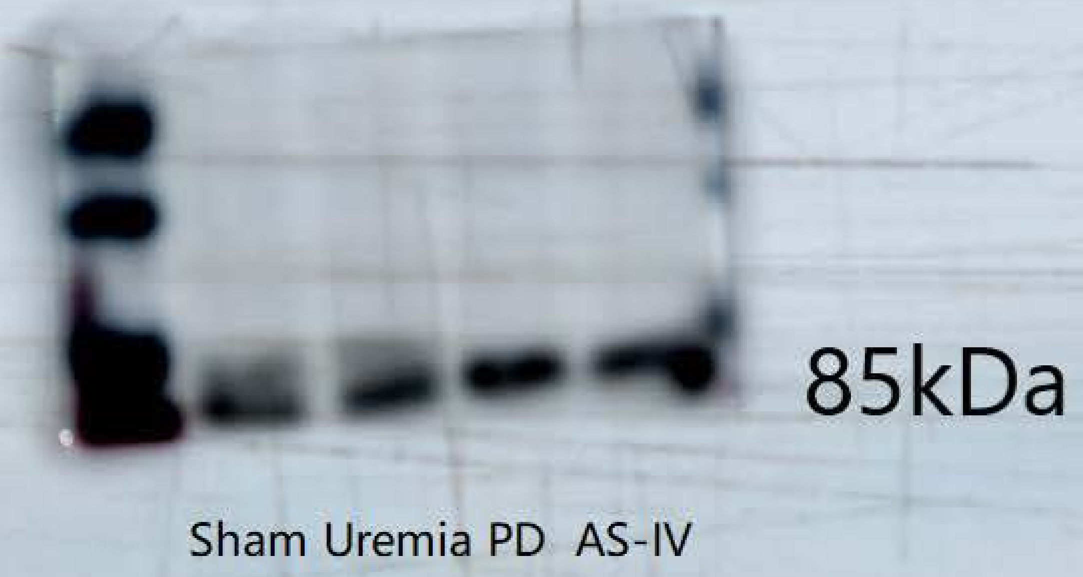

Fig 2J PI3K

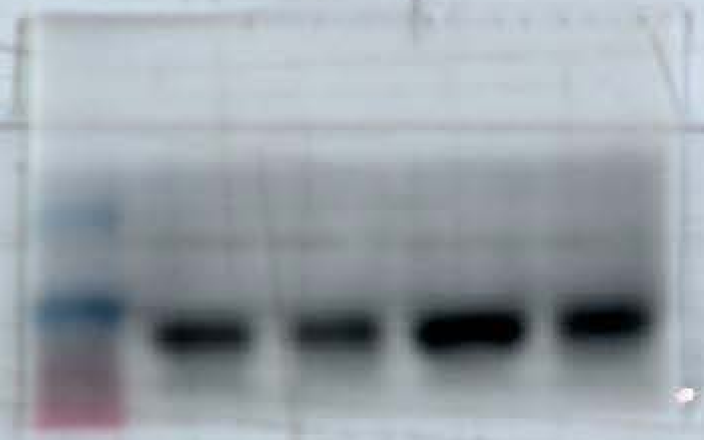

Fig 2J pPI3K

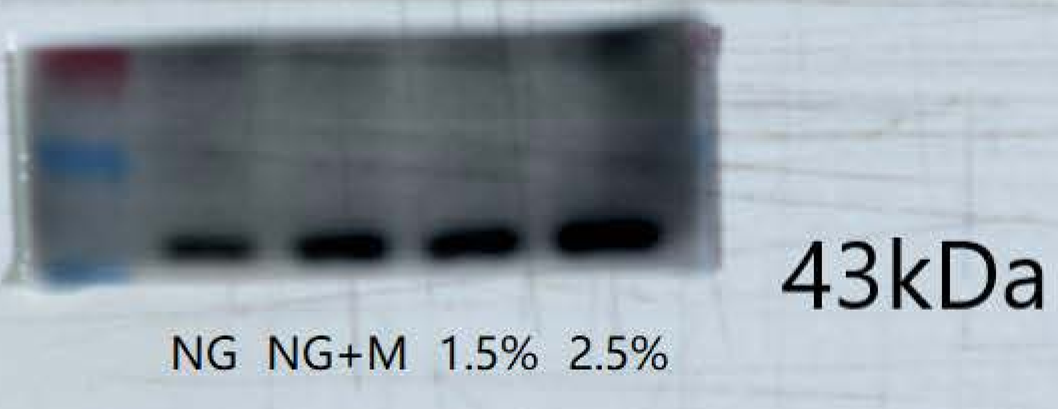

Fig 3A ASMA

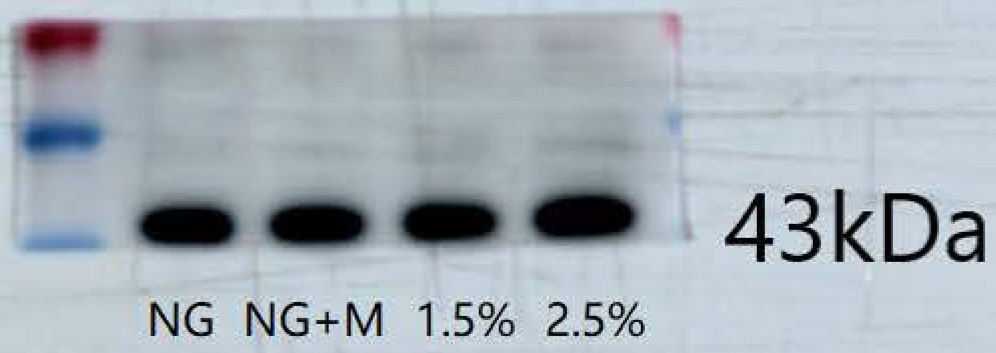

Fig 3A BACTIN

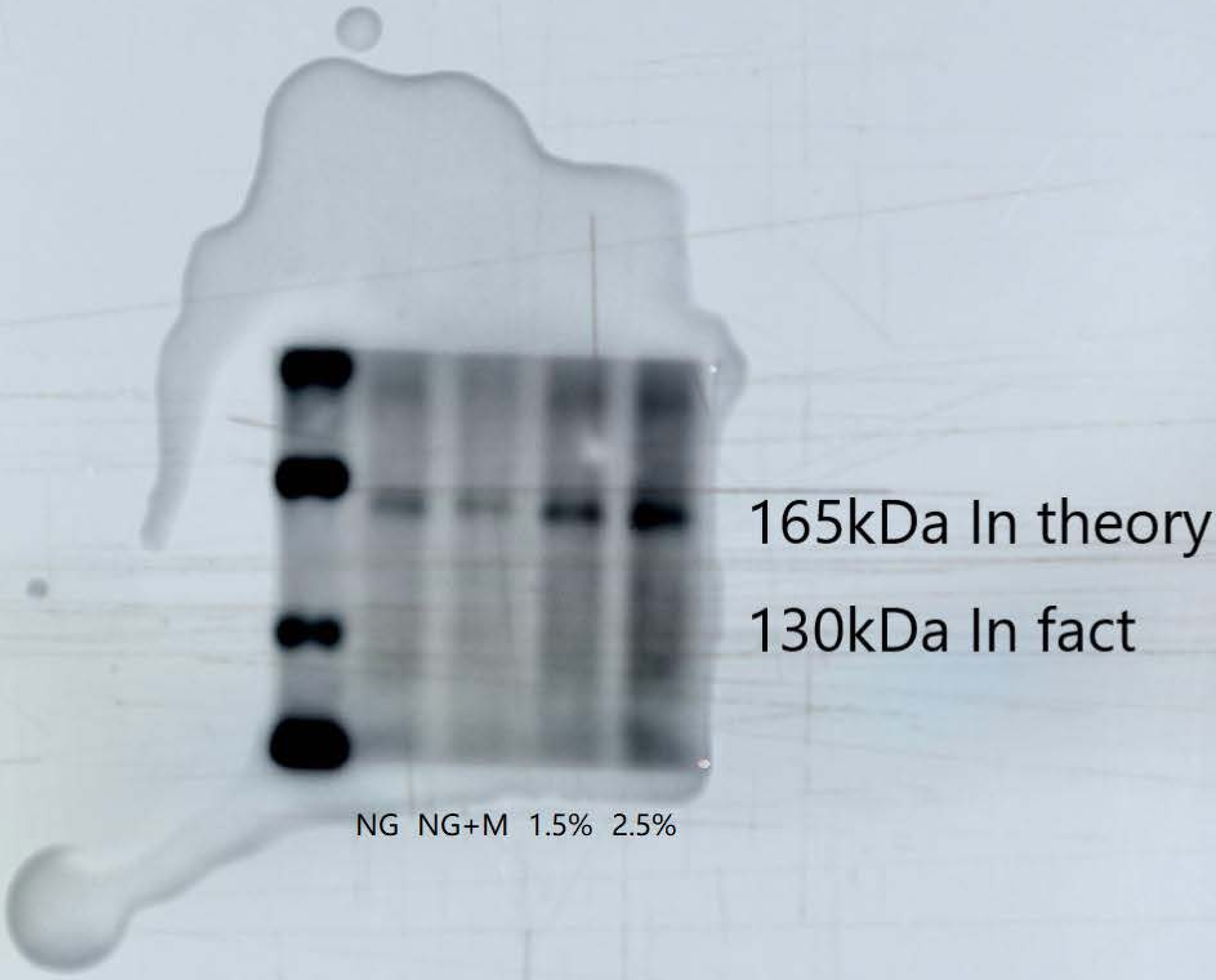

Fig 3A Col-IV

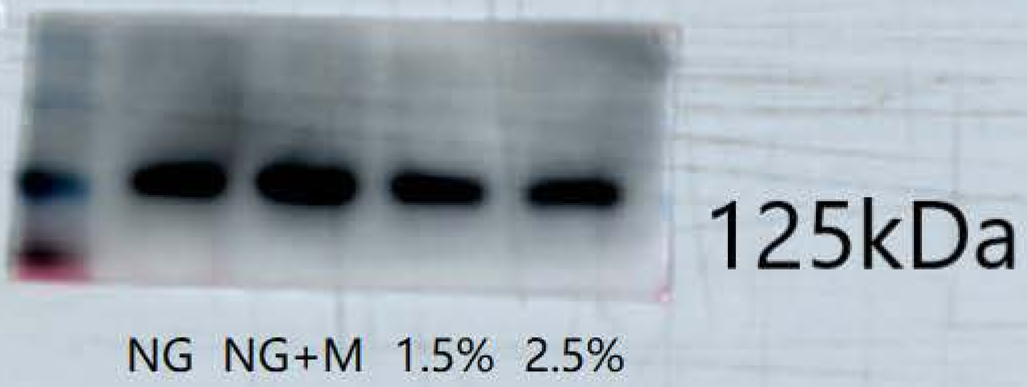

Fig 3A E-cadherin

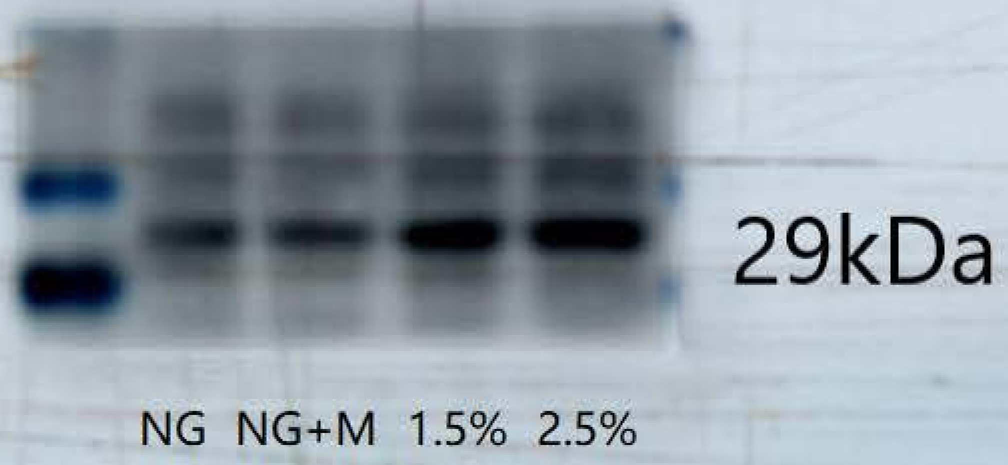

Fig 3A ENKUR

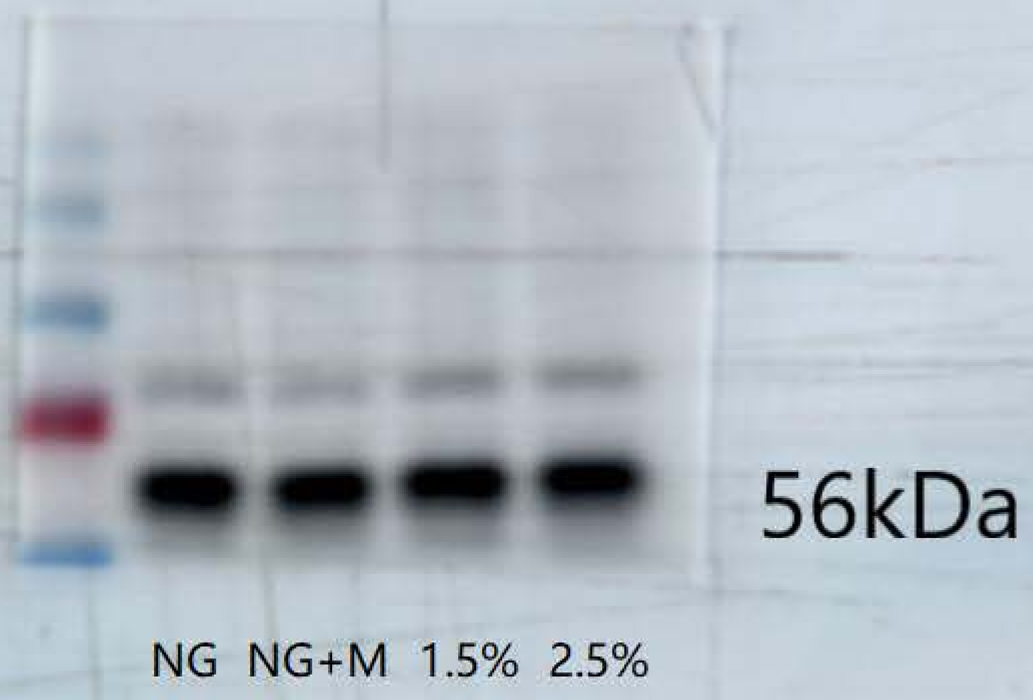

Fig 3J AKT

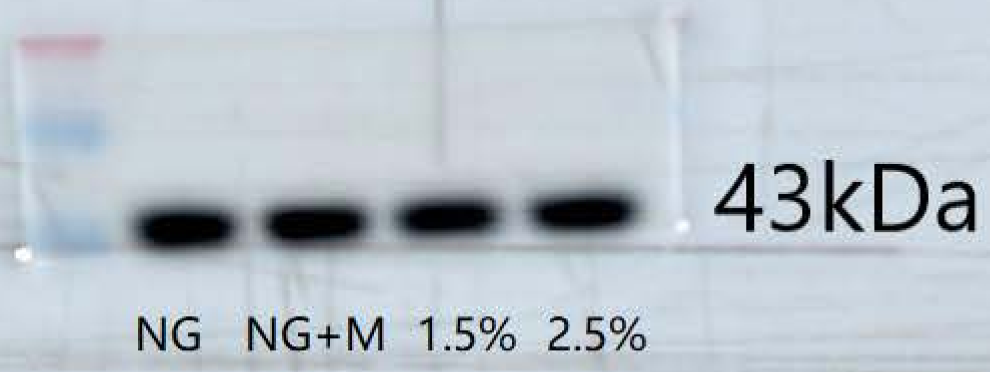

Fig 3J BACTIN

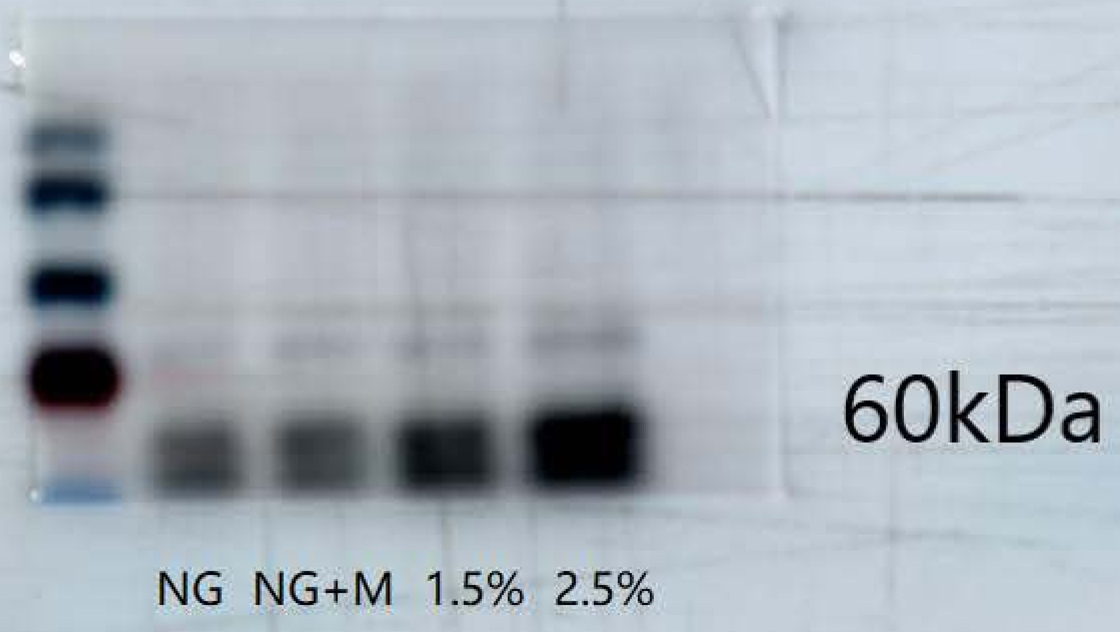

Fig 3J pAKT

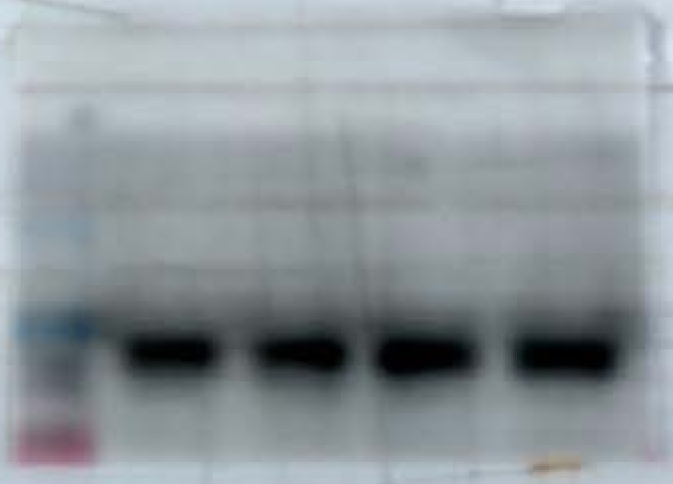

Fig 3J PI3K

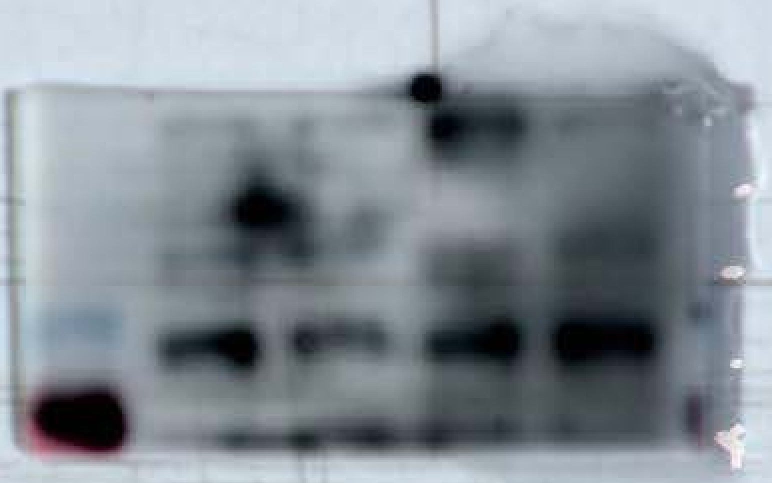

Fig 3J pPI3K

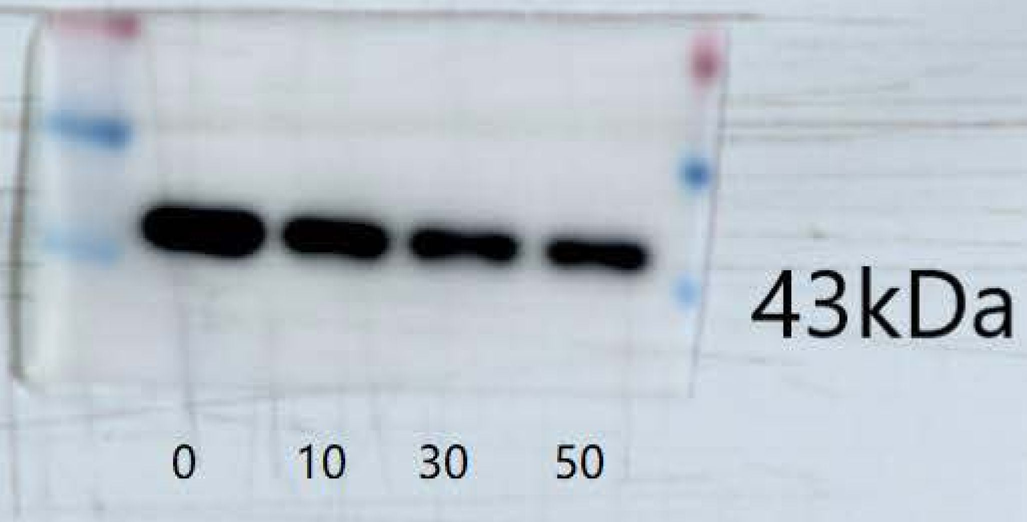

Fig 4A ASMA

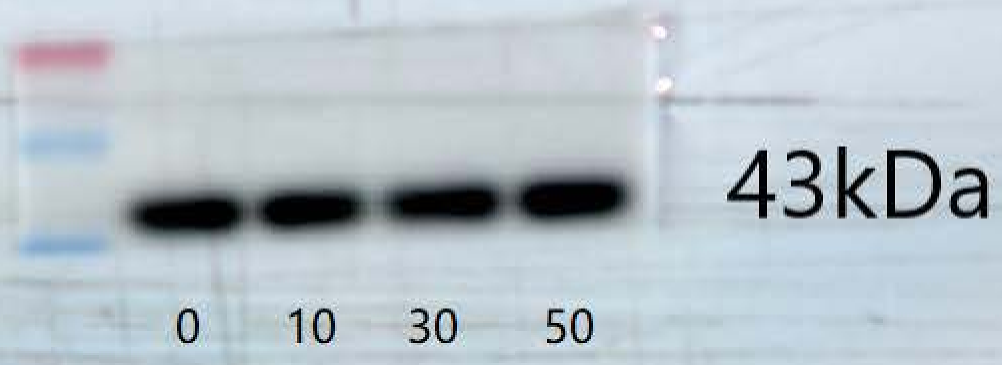

Fig 4A BACTIN

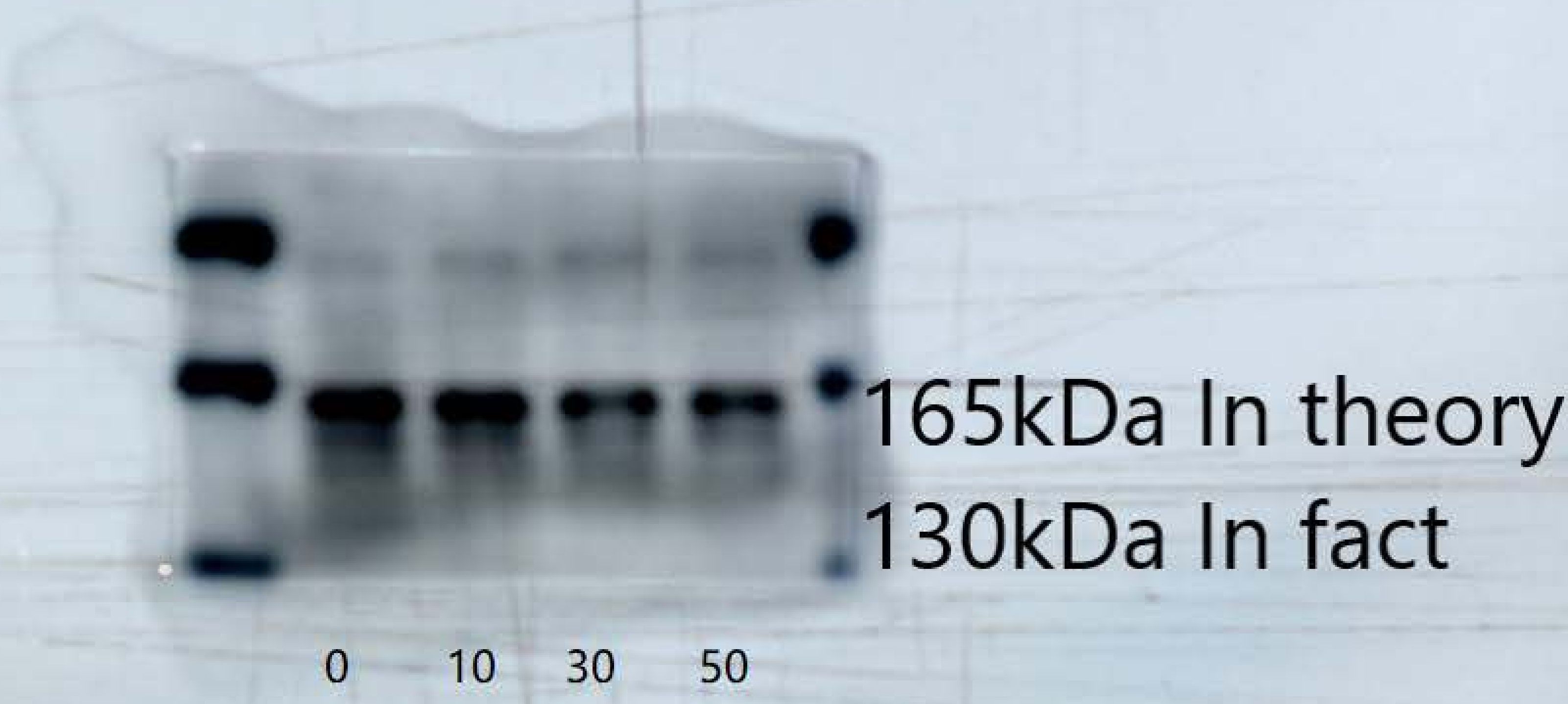

Fig 4A Col-IV

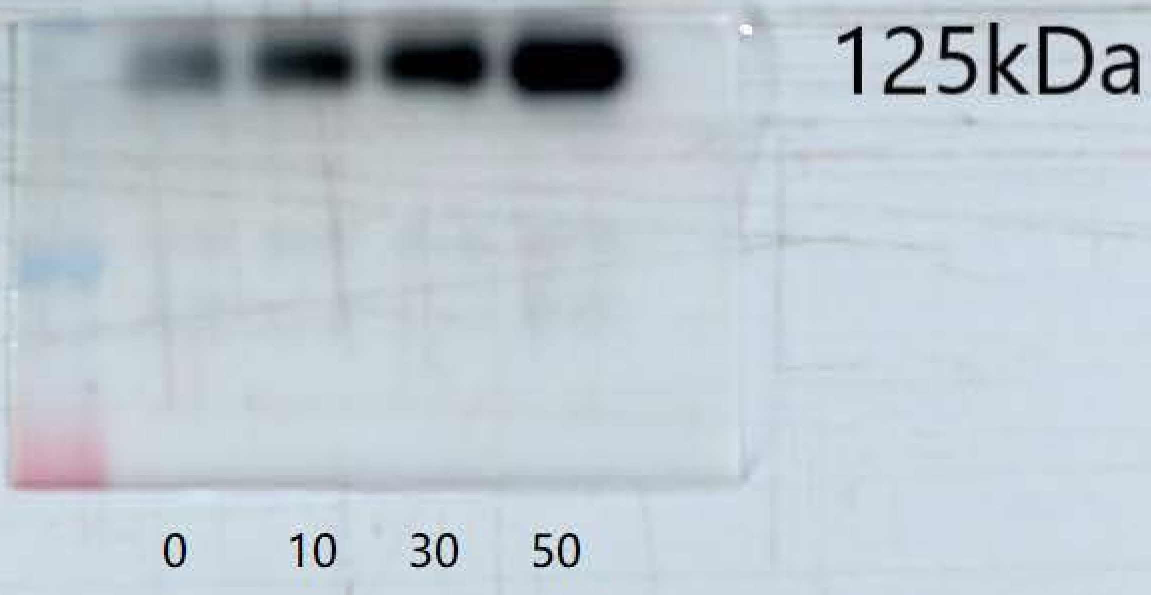

Fig 4A E-cadherin

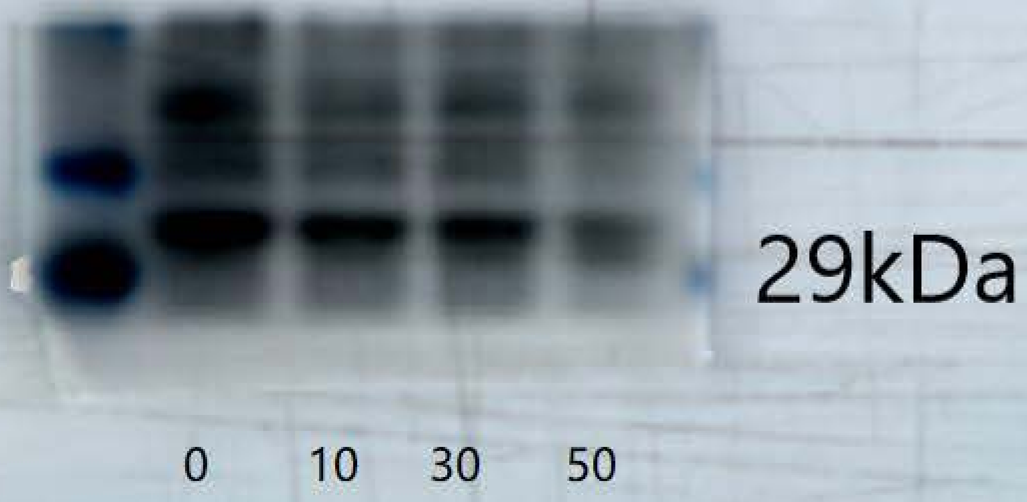

Fig 4A ENKUR

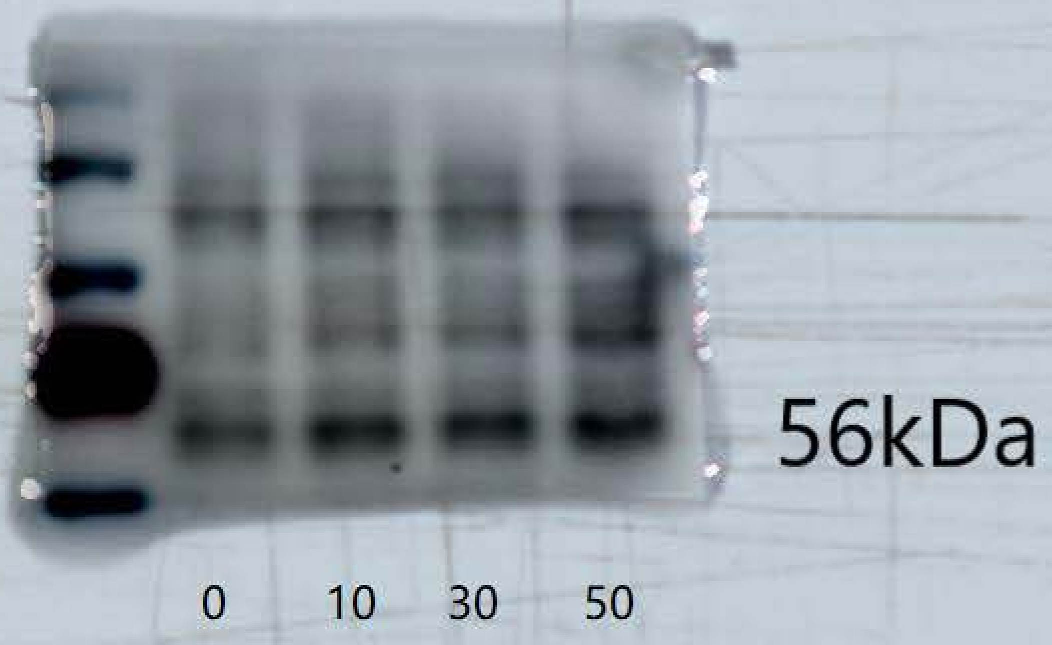

Fig 4J AKT

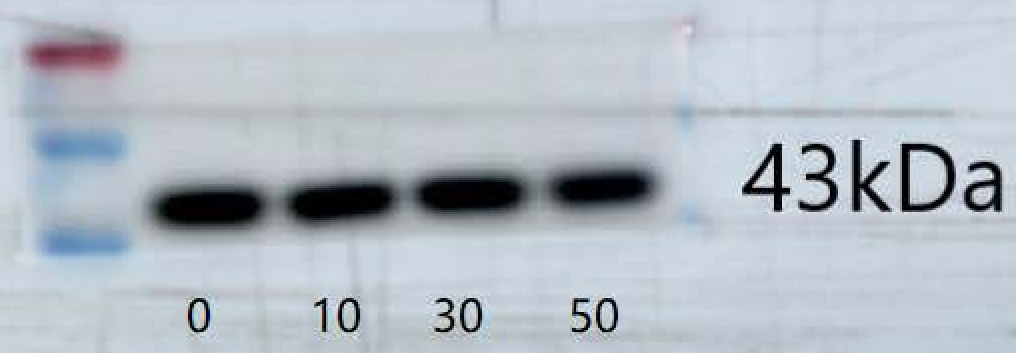

Fig 4J BACTIN

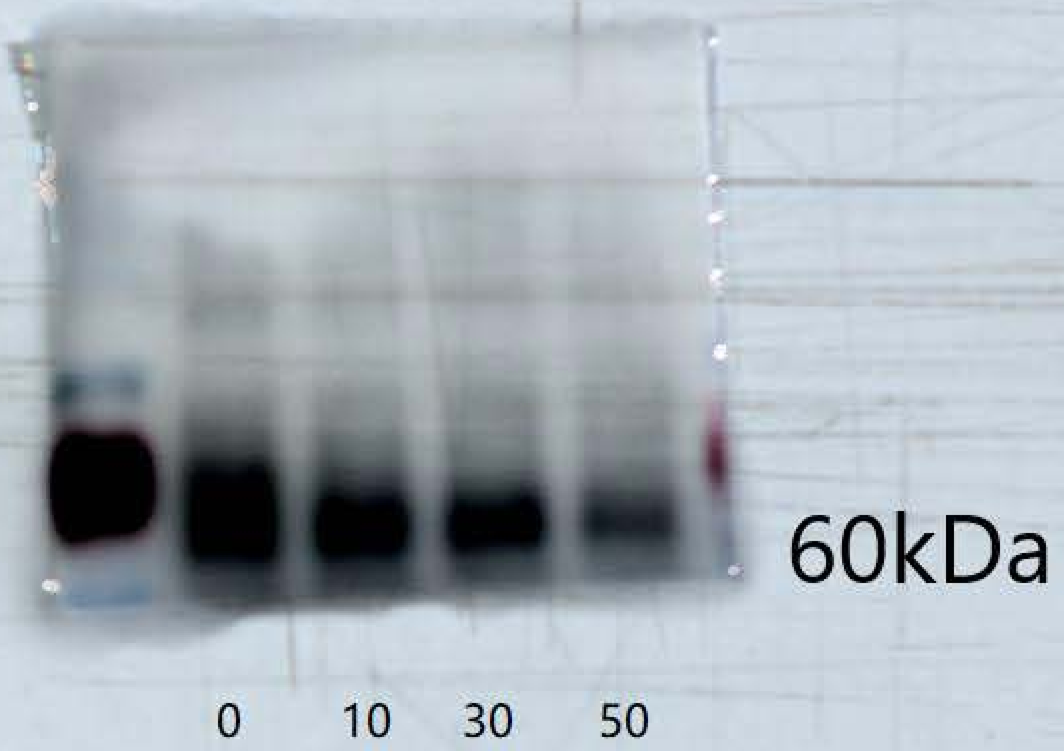

Fig 4J pAKT

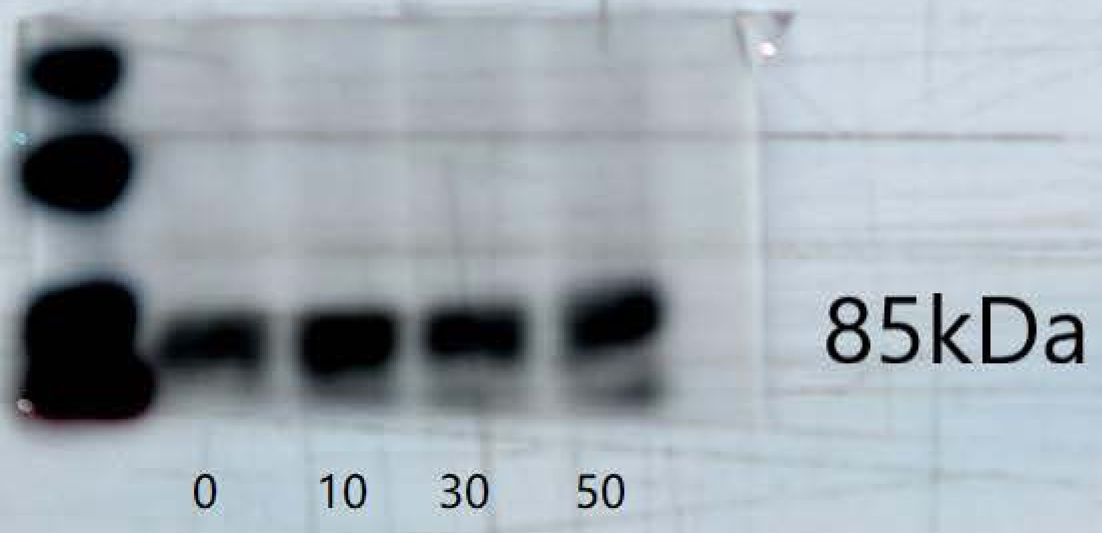

Fig 4J PI3K

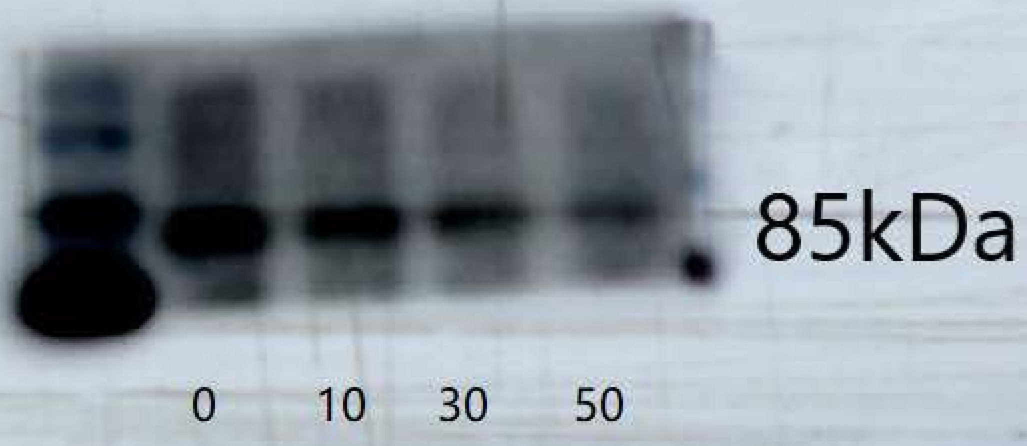

Fig 4J pPI3K
