# Supplementary material for: Astragaloside IV attenuates high-glucose-Induced peritoneal fibrosis via modulation of the ENKUR/PI3K/Akt signalling pathway
Source: PLoS One. 2026 May 8;21(5):e0348762. doi: 10.1371/journal.pone.0348762 (PMC13155615; doi:10.1371/journal.pone.0348762)
Supplement: S3 File — (PDF) [file pone.0348762.s003.pdf]

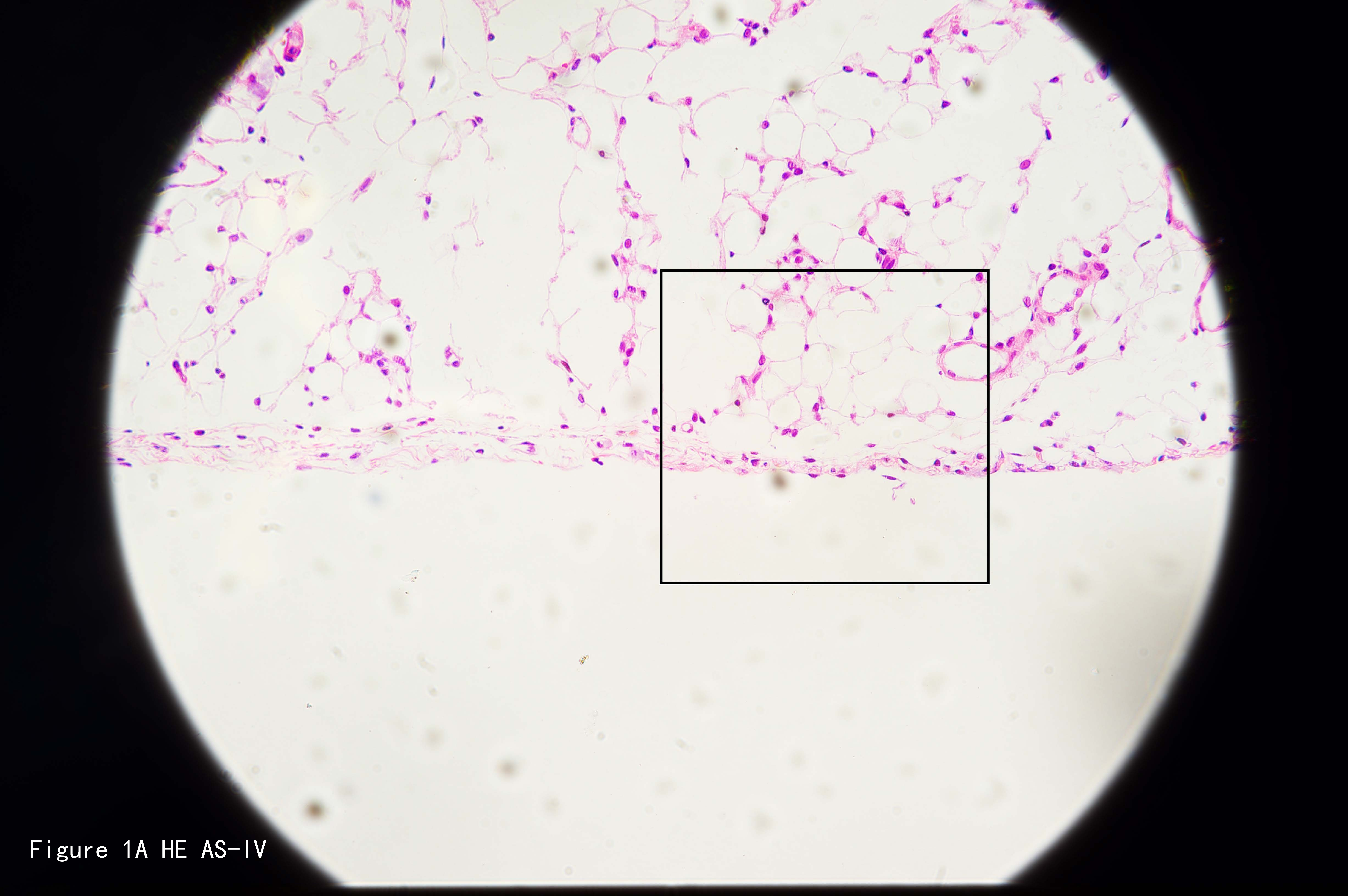

Figure 1A HE AS-IV

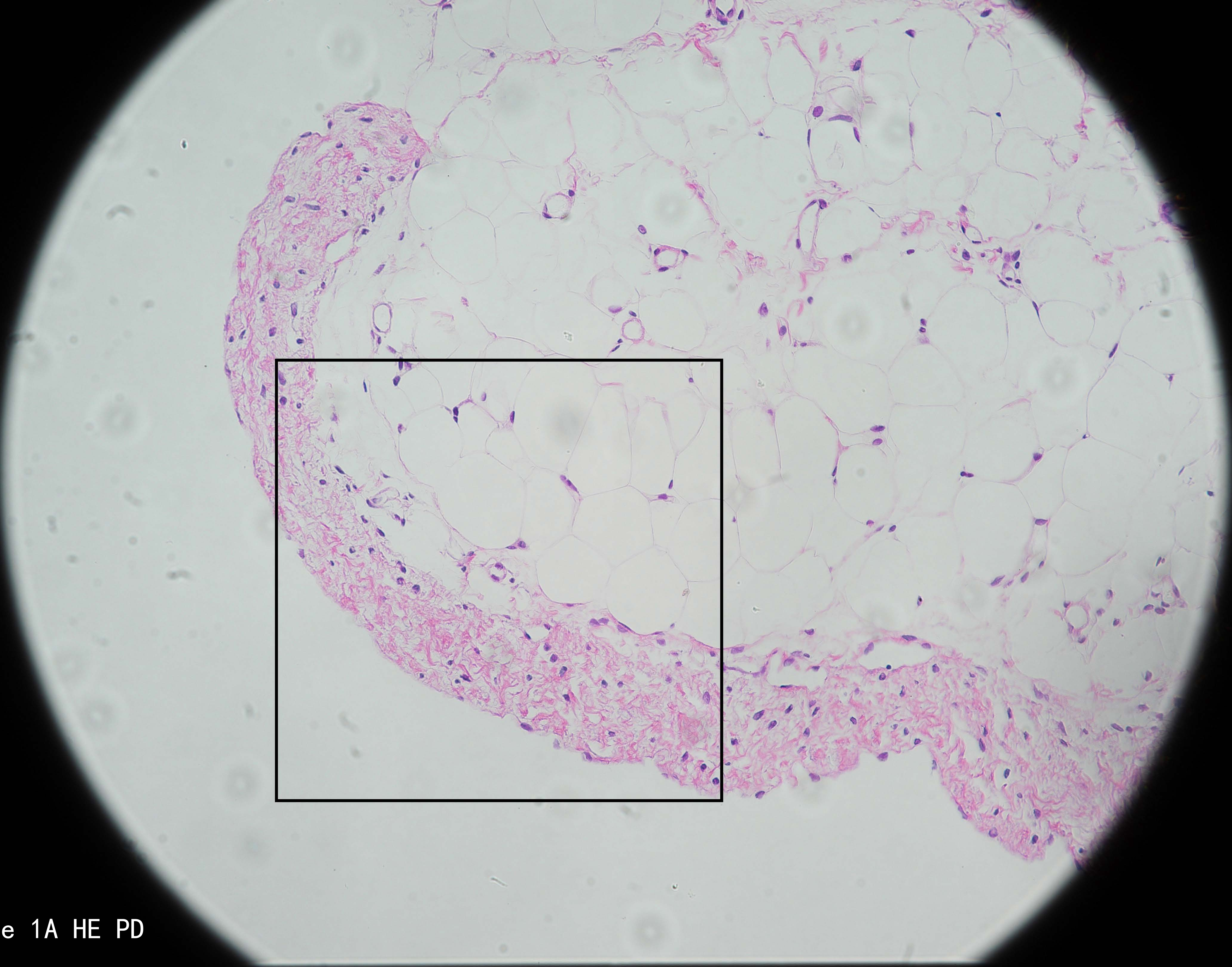
Figure 1A HE PD

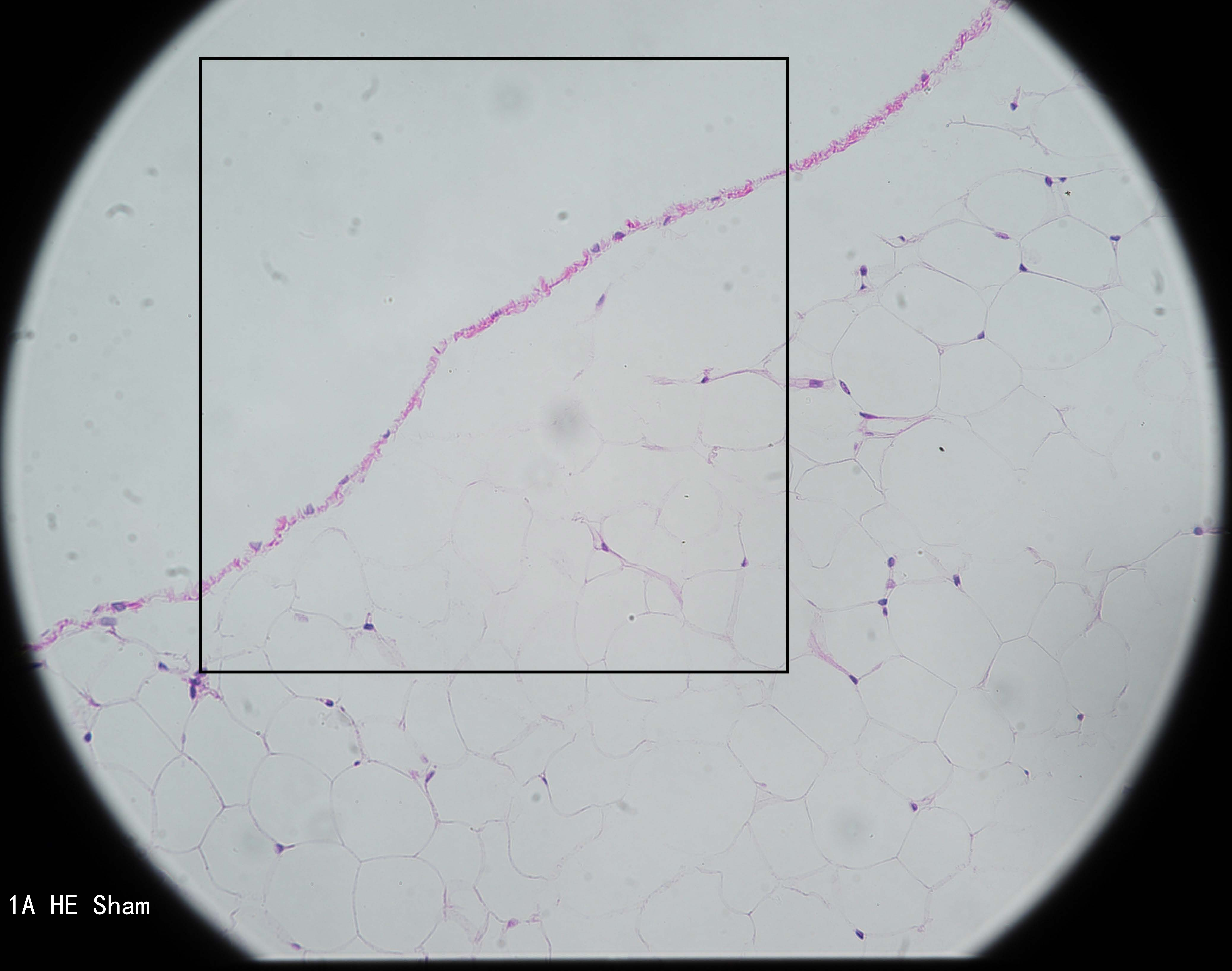

Figure 1A HE Sham

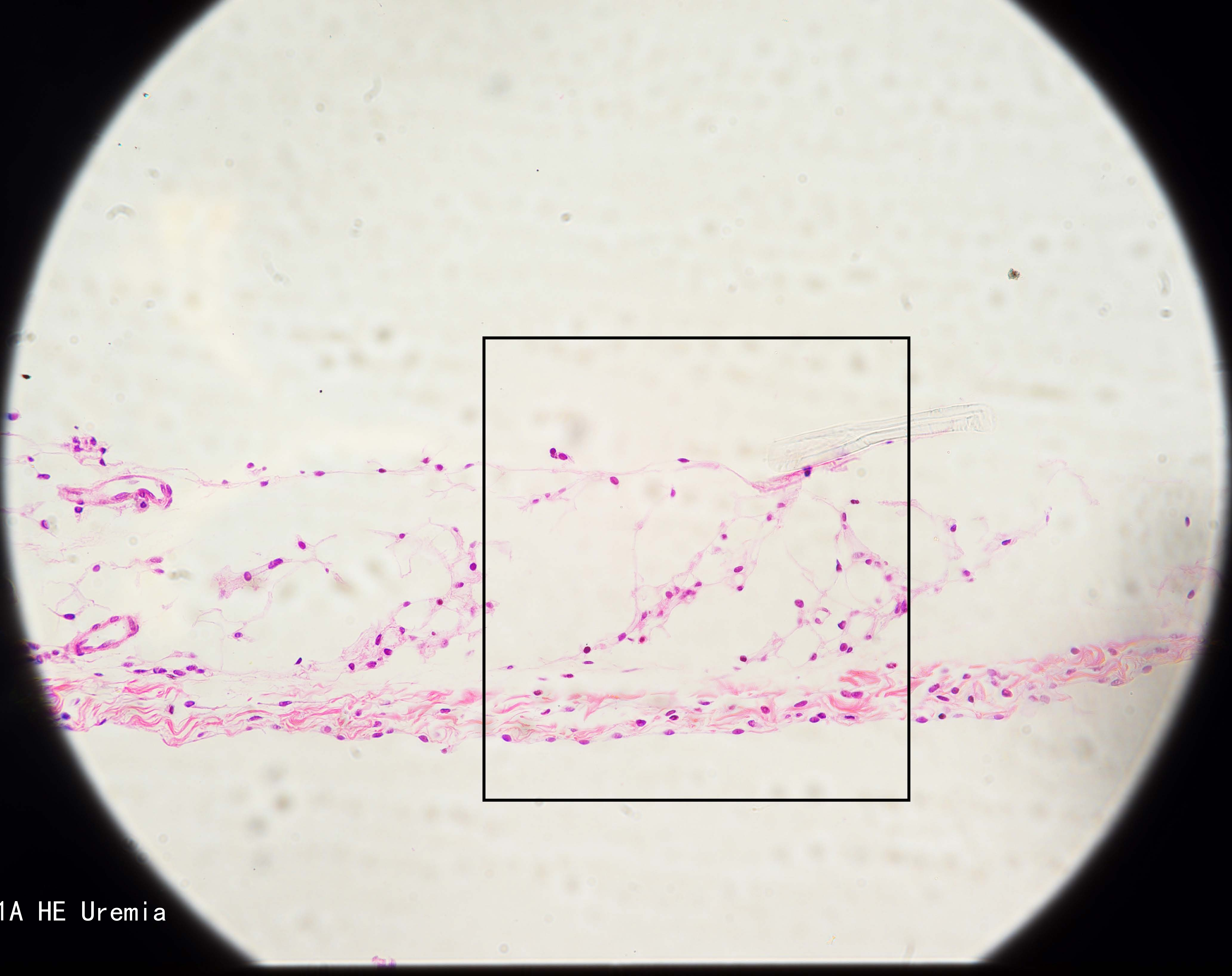

Figure 1A HE Uremia

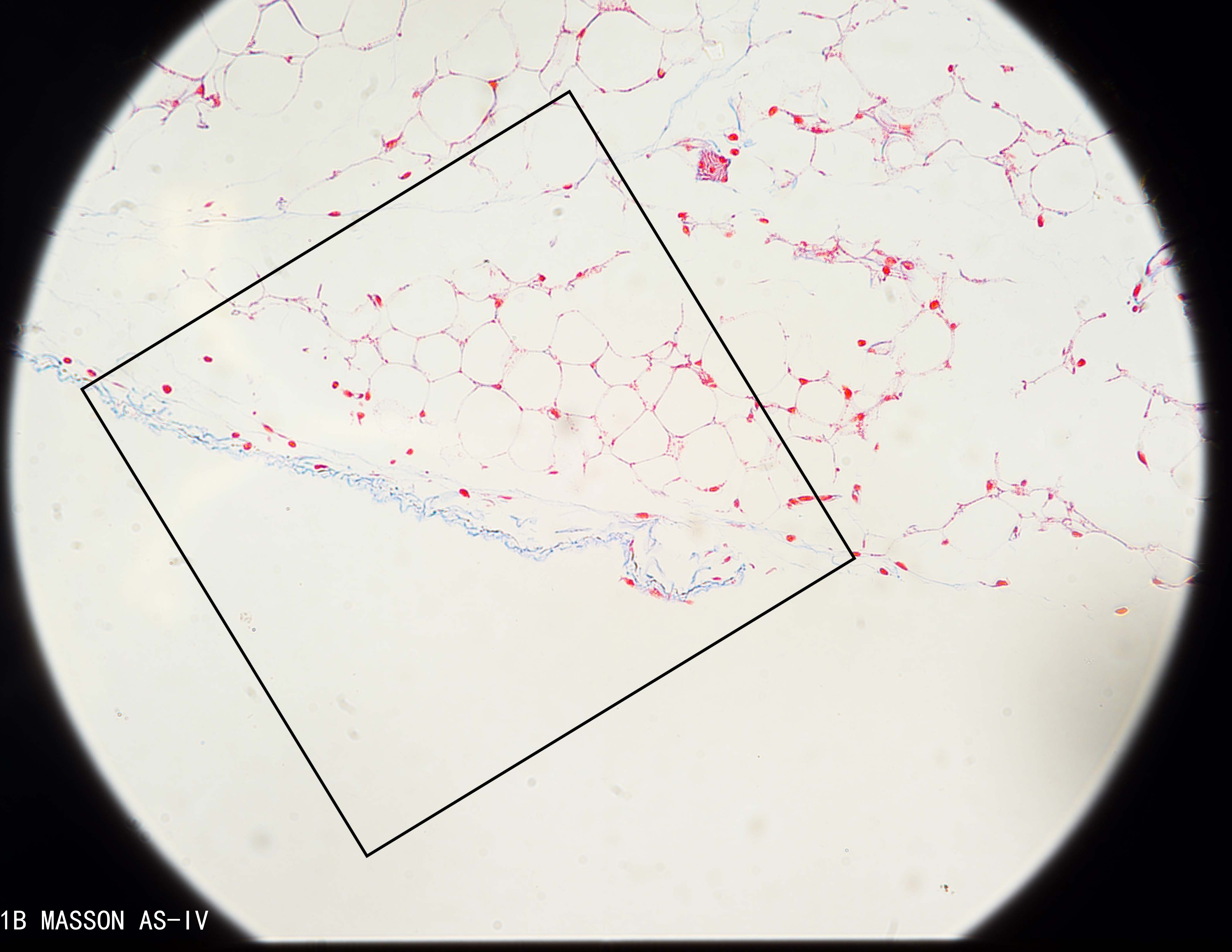

Figure 1B MASSON AS-IV

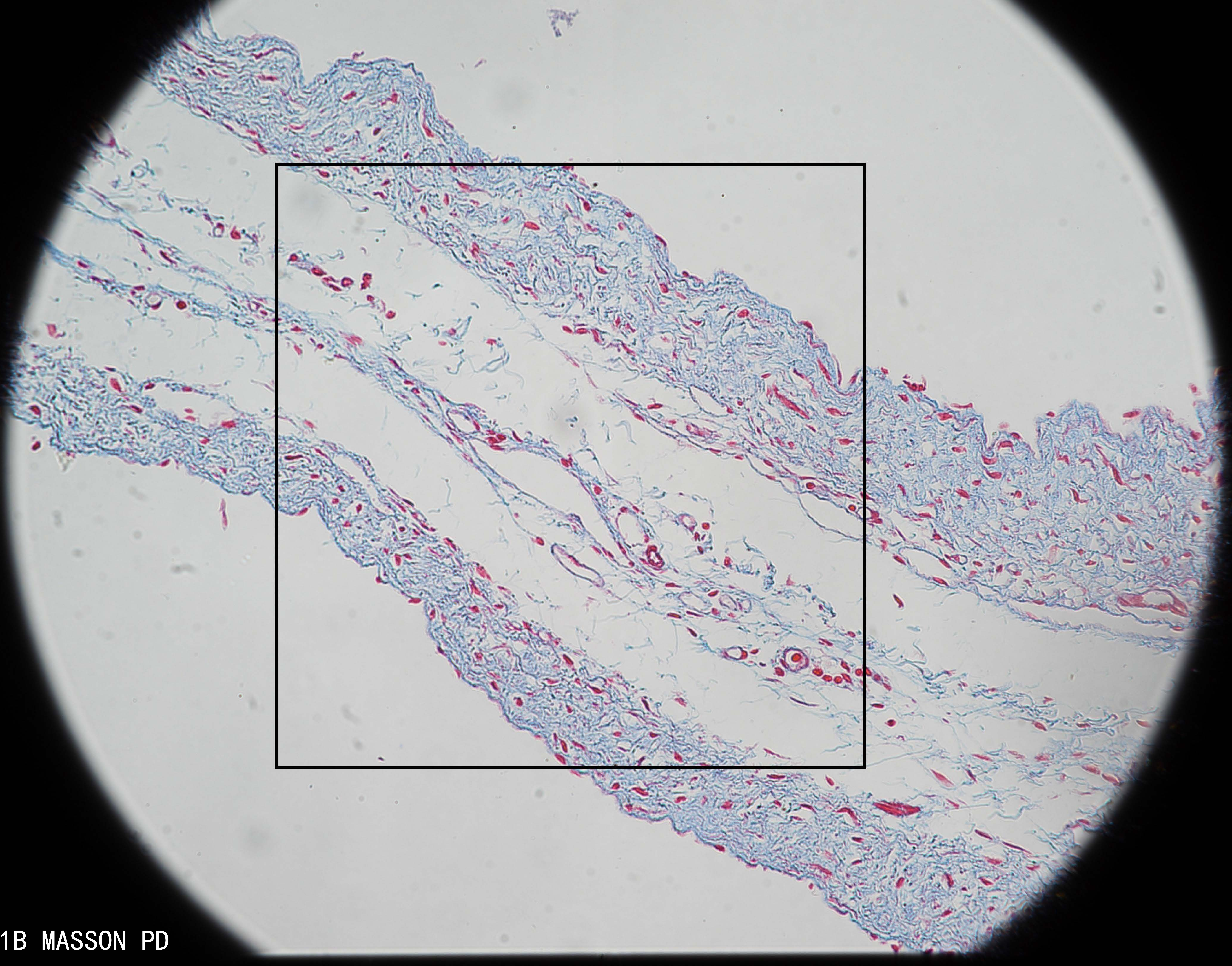

Figure 1B MASSON PD

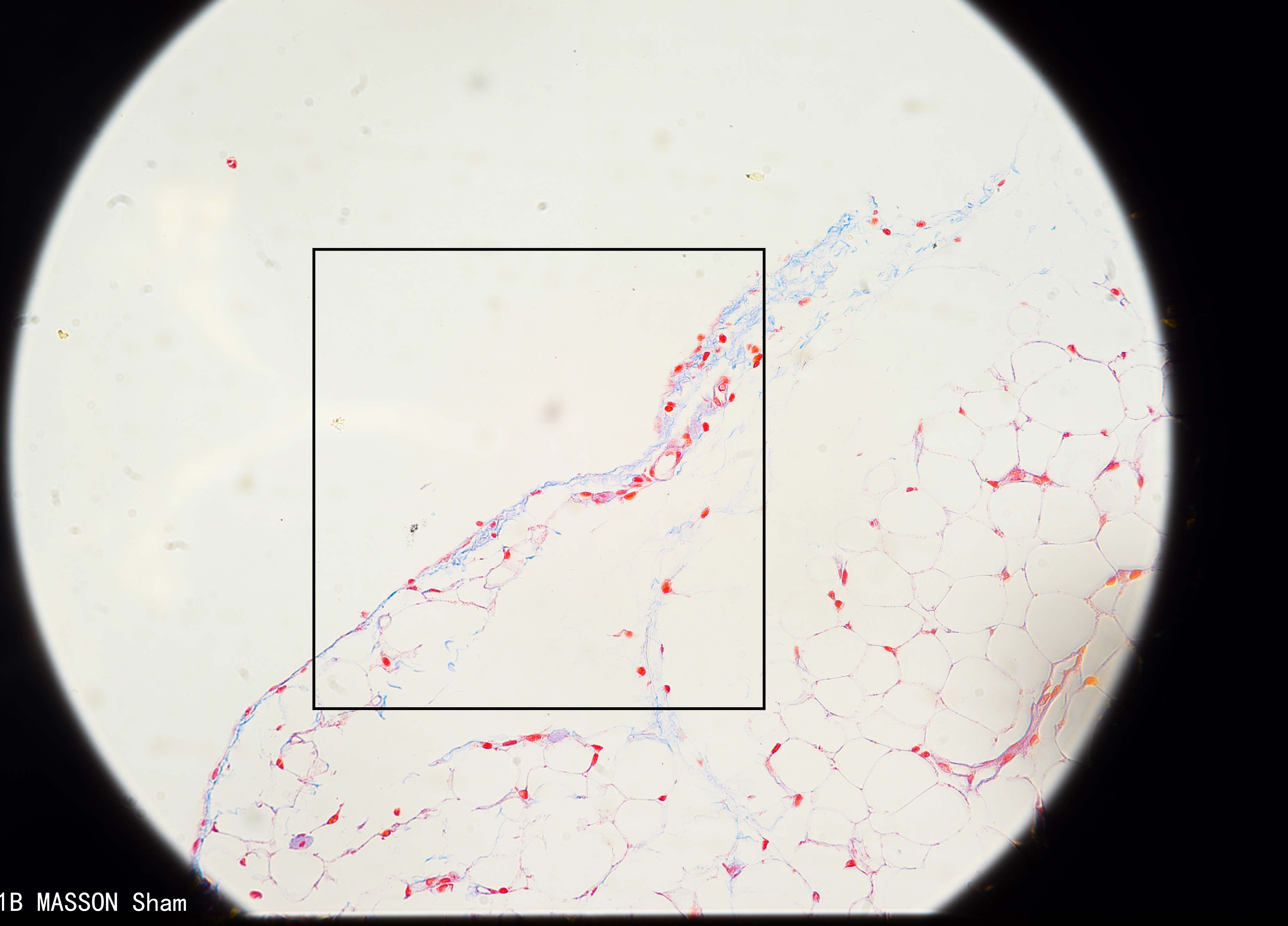

Figure 1B MASSON Sham

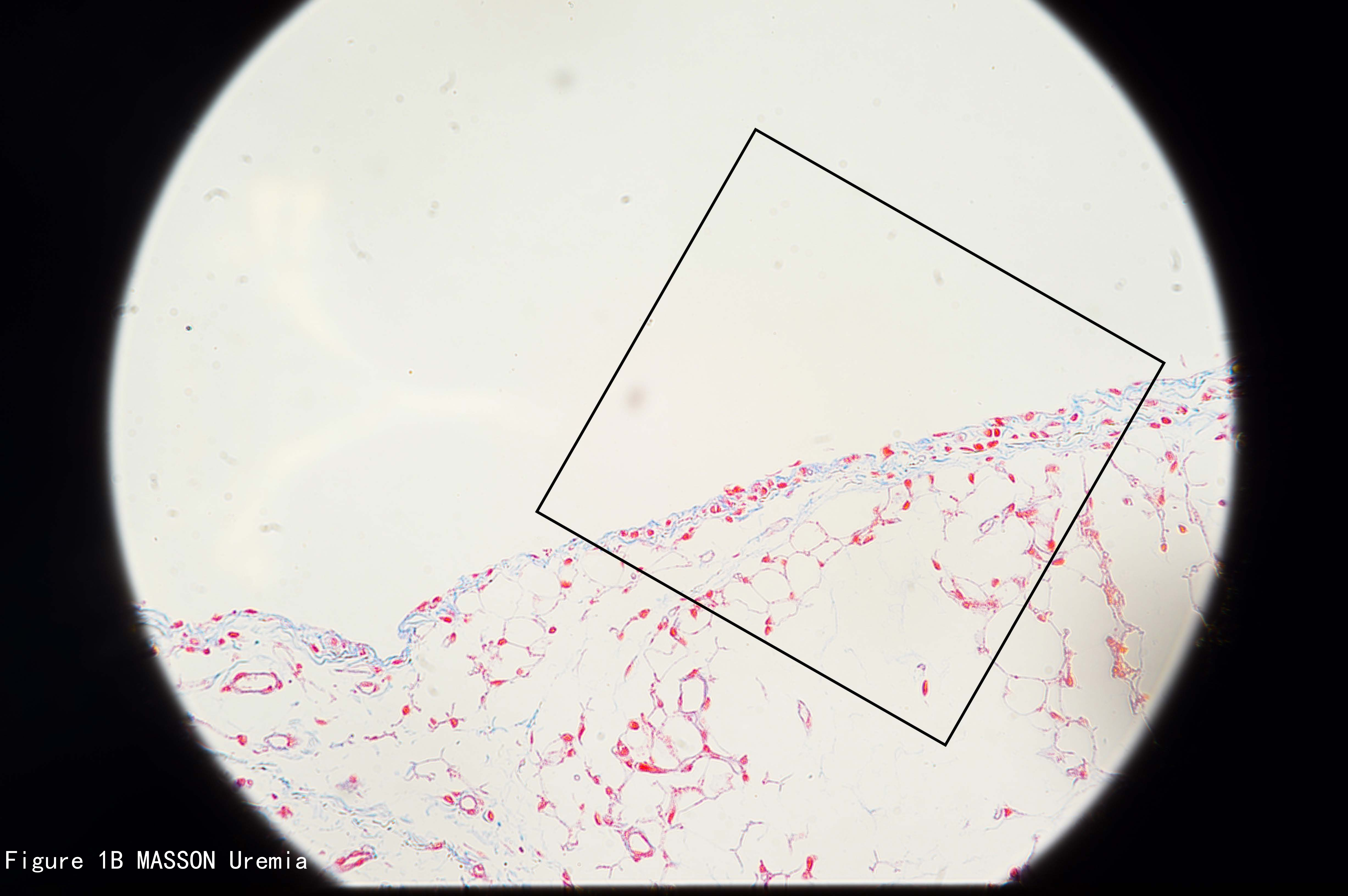

Figure 1B MASSON Uremia

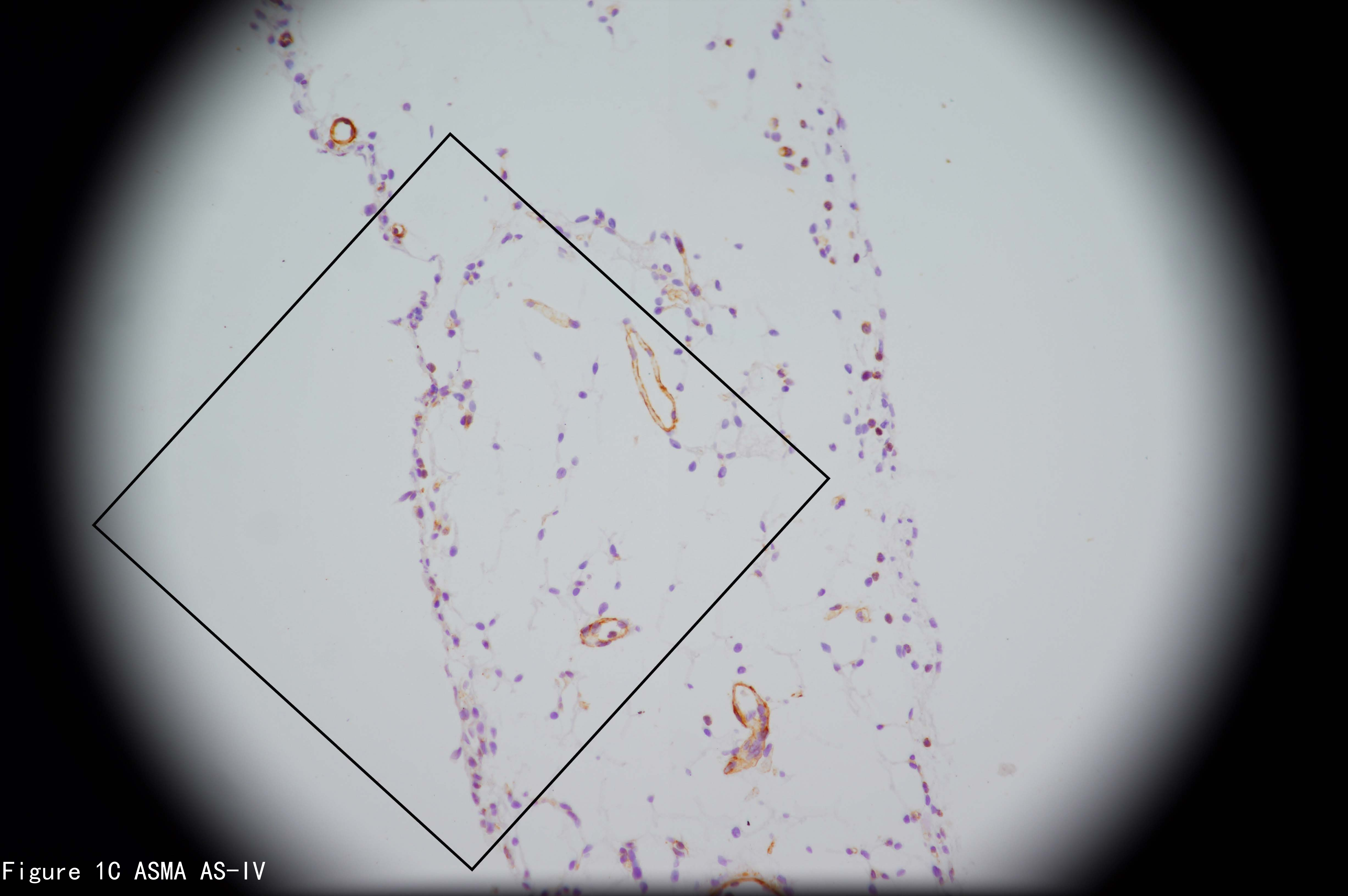

Figure 1C ASMA AS-IV

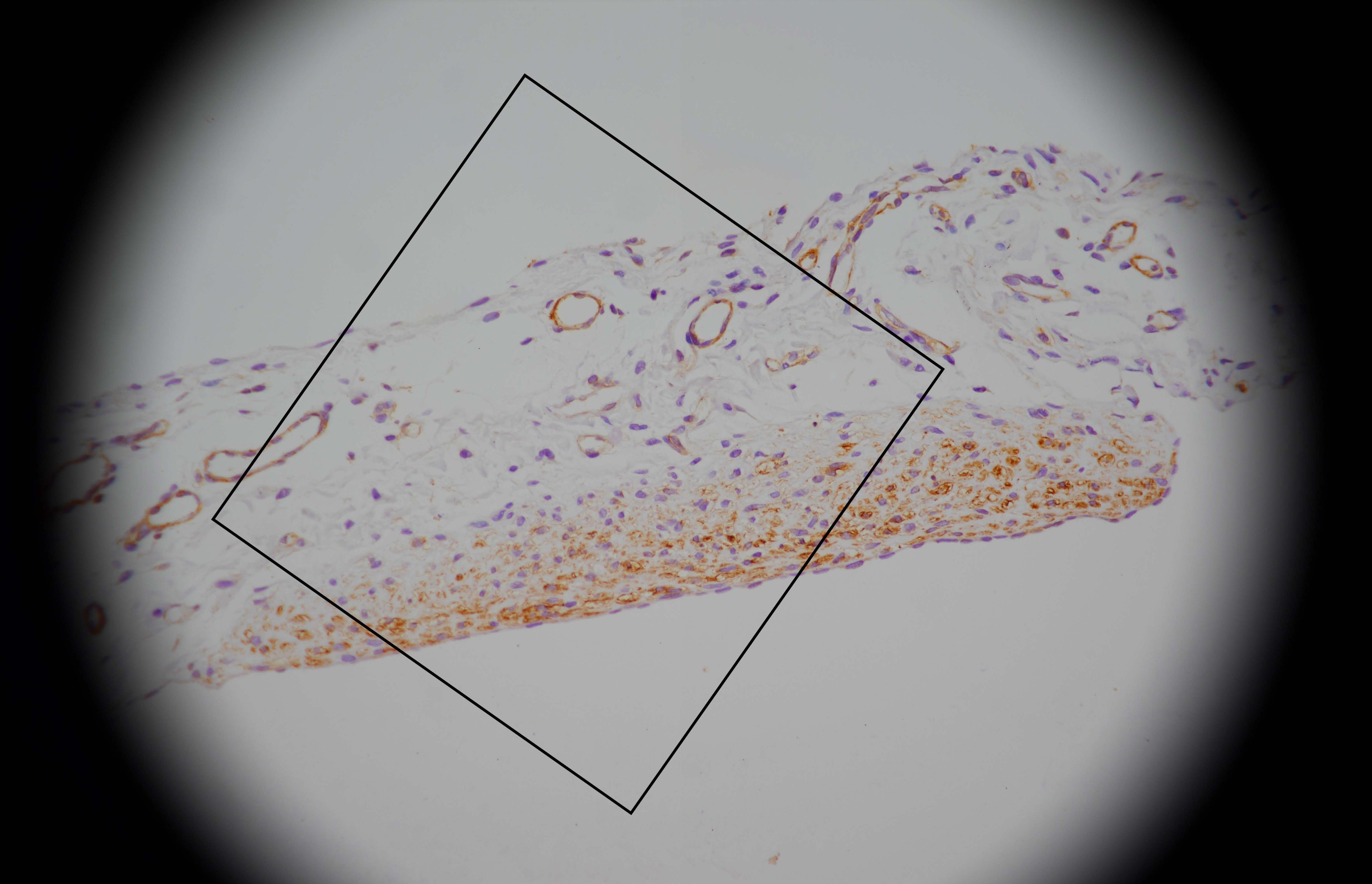

Figure 1C ASMA PD

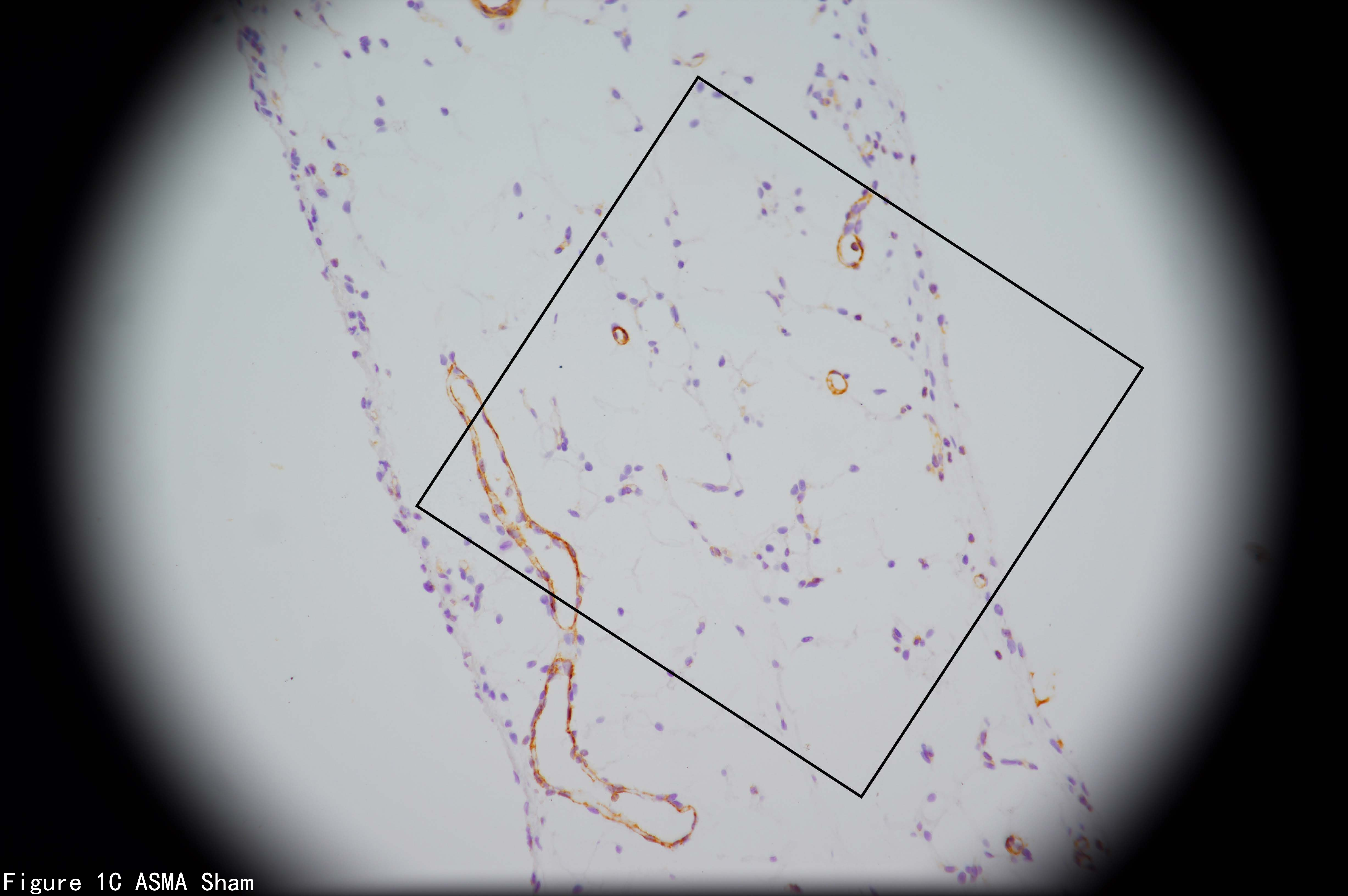

Figure 1C ASMA Sham

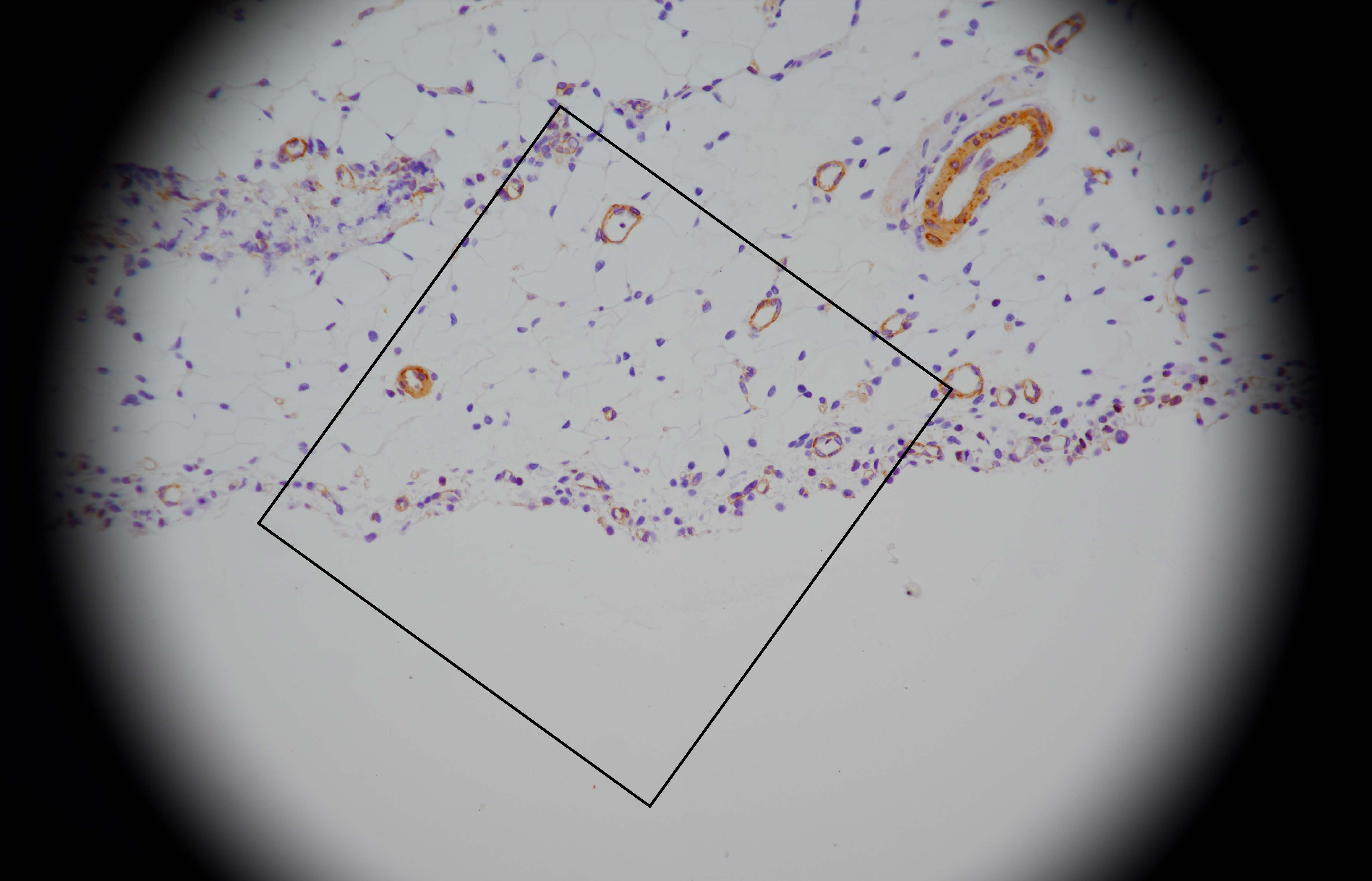

Figure 1C ASMA Uremia

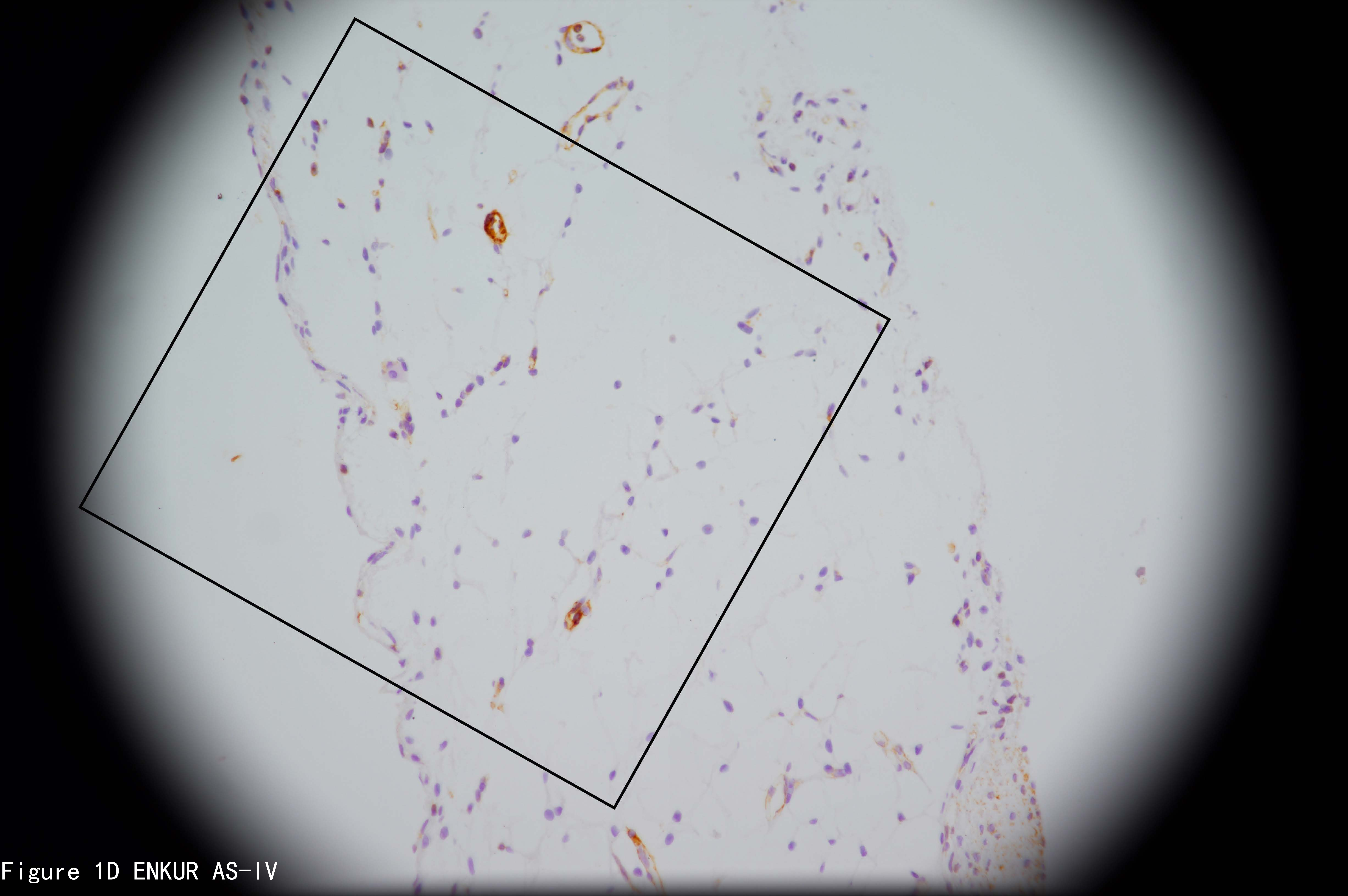

Figure 1D ENKUR AS-IV

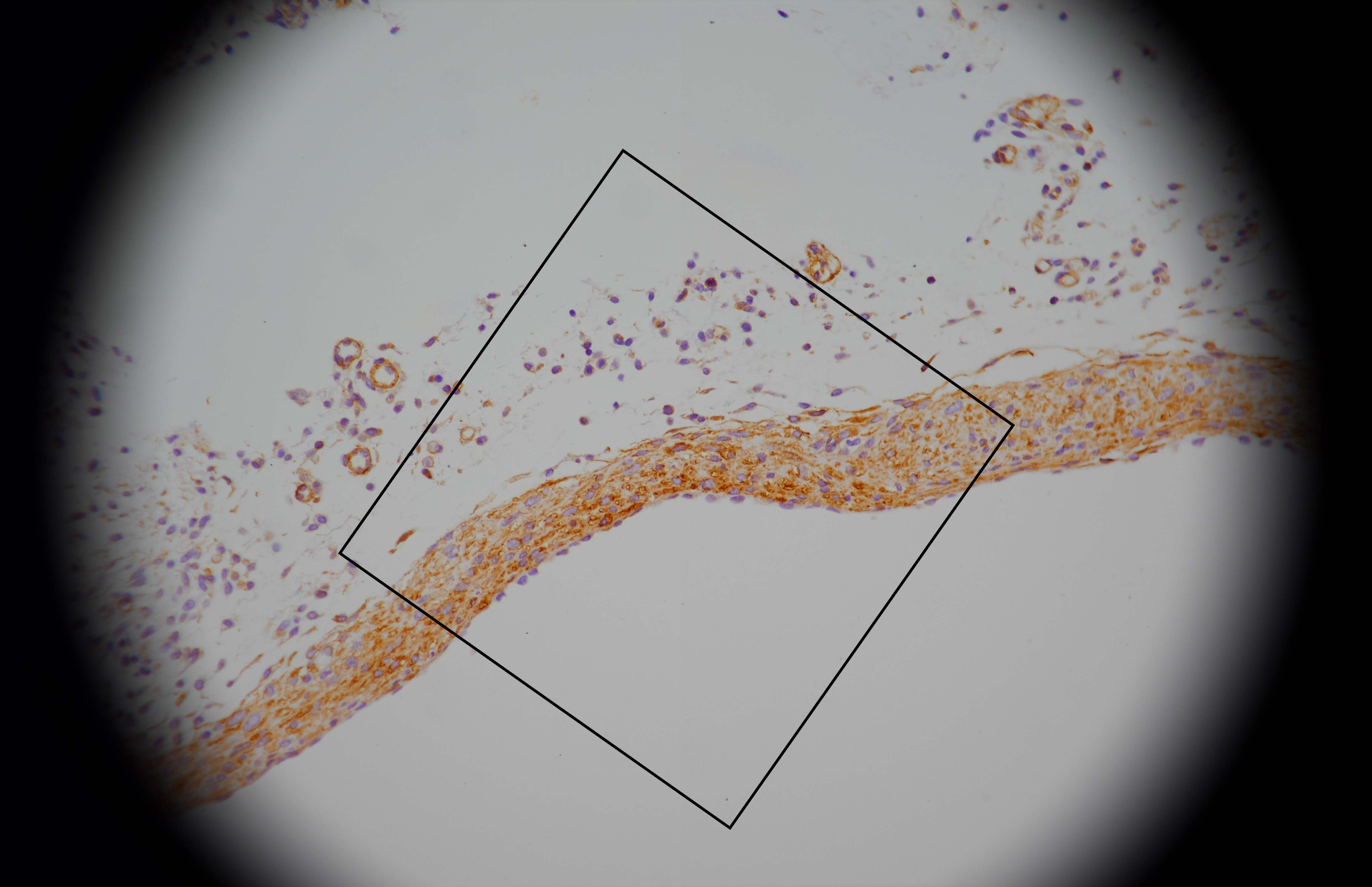

Figure 1D ENKUR PD

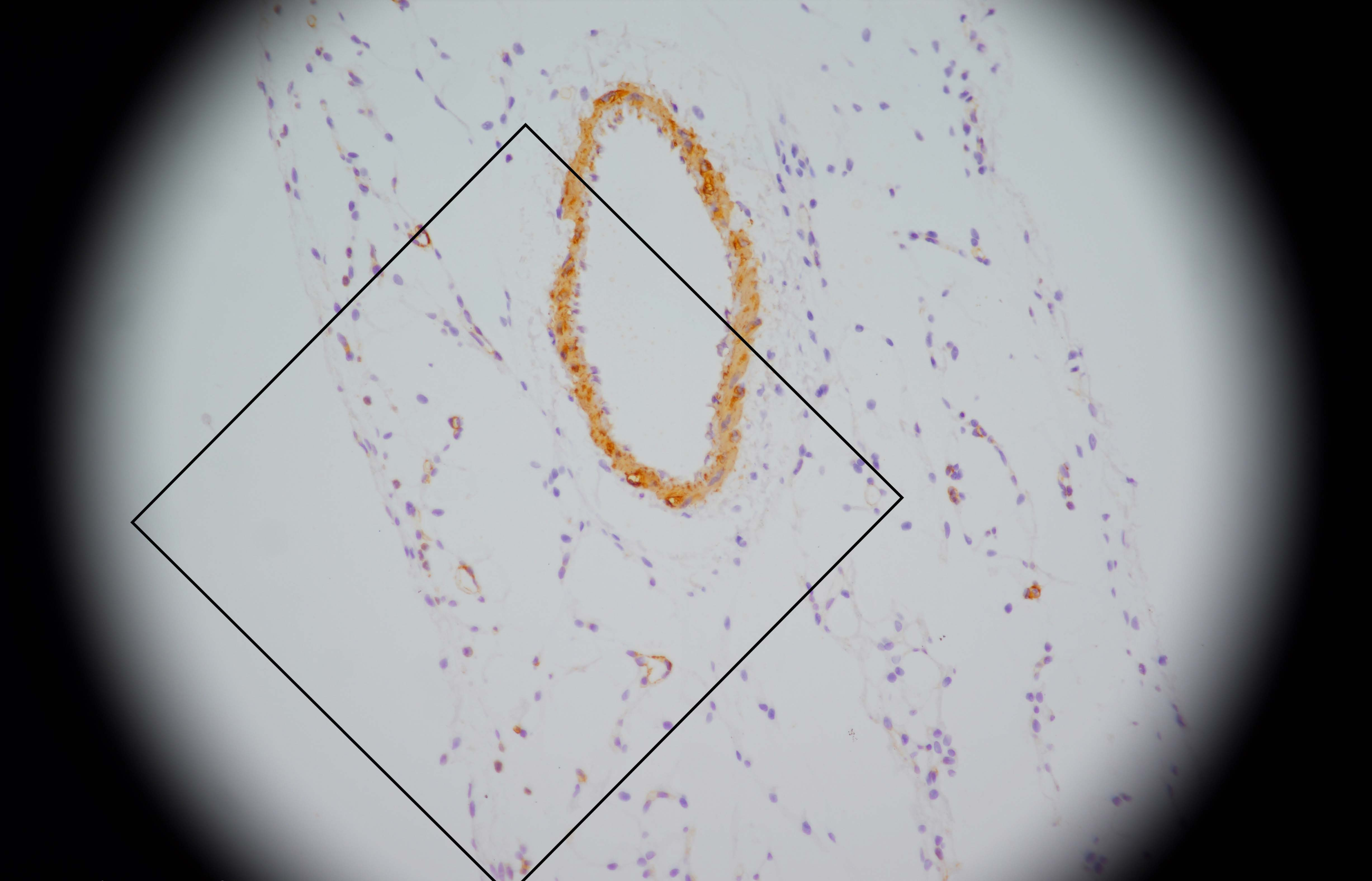

Figure 1D ENKUR Sham

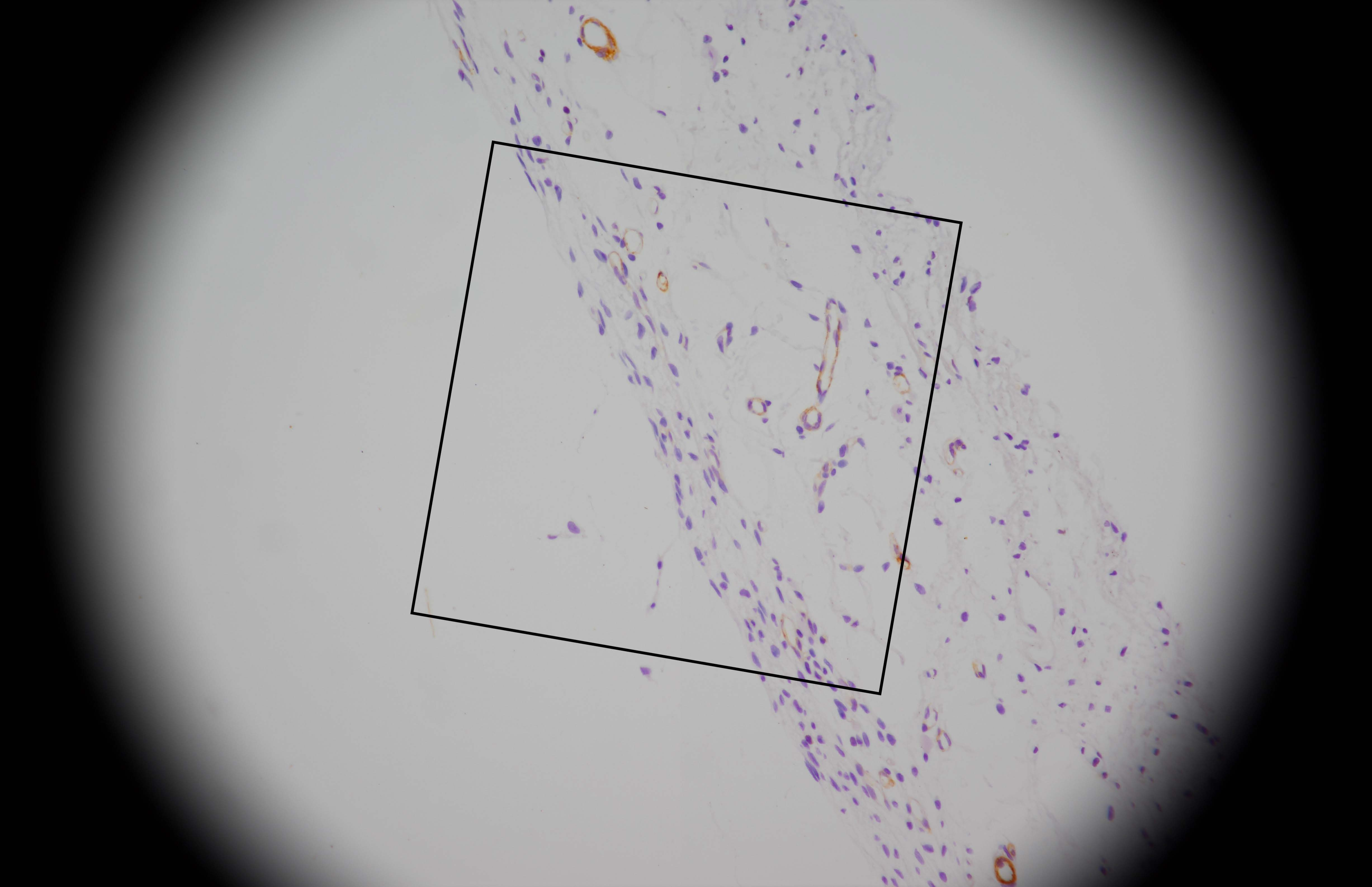

Figure 1D ENKUR Uremia
